# Targeting SPHK1/S1PR3-regulated S-1-P metabolic disorder triggers autophagic cell death in pulmonary lymphangiomyomatosis (LAM)

**DOI:** 10.1038/s41419-022-05511-3

**Published:** 2022-12-21

**Authors:** Fei Li, Yifan Zhang, Zhoujun Lin, Lizhong Yan, Qiao Liu, Yin Li, Xiaolin Pei, Ya Feng, Xiao Han, Juan Yang, Fangxu Zheng, Tianjiao Li, Yupeng Zhang, Zhenkun Fu, Di Shao, Jane Yu, Chenggang Li

**Affiliations:** 1 State Key Laboratory of Medicinal Chemical Biology and College of Pharmacy, Nankai University, 300350 Tianjin, P.R. China; 2 Department of Immunology & Wu Lien-Teh Institute & Heilongjiang Provincial Key Laboratory for Infection and Immunity, Harbin Medical University & Heilongjiang Academy of Medical Science, Harbin, China; 3 Chongqing University Central Hospital, Chongqing Emergency Medical Center, 400000 Chongqing, China; 4 Chonggang General Hospital, 400000 Chongqing, China; 5 Division of Pulmonary, Critical Care, and Sleep Medicine, Department of Internal Medicine, University of Cincinnati College of Medicine, Cincinnati, OH 45267 USA

**Keywords:** Cancer metabolism, Autophagy

## Abstract

Lymphangioleiomyomatosis (LAM), a progressive pulmonary disease exclusively affecting females, is caused by defects or mutations in the coding gene tuberous sclerosis complex 1 (*TSC1)* or *TSC2*, causing the mammalian target of rapamycin complex 1 (mTORC1) activation and autophagy inhibition. Clinically, rapamycin shows limited cytocidal effects, and LAM recurs after drug withdrawal. In this study, we demonstrated that TSC2 negatively regulated the sphingolipid metabolism pathway and the expressions of sphingosine kinase 1 (SPHK1) and sphingosine-1-phosphate receptor 3 (S1PR3) were significantly elevated in LAM patient-derived TSC2-deficient cells compared to TSC2-addback cells, insensitive to rapamycin treatment and estrogen stimulation. Knockdown of SPHK1 showed reduced viability, migration and invasion in TSC2-deficient cells. Selective SPHK1 antagonist PF543 potently suppressed the viability of TSC2-deficient cells and induced autophagy-mediated cell death. Meanwhile, the cognate receptor S1PR3 was identified to mediating the tumorigenic effects of sphingosine-1-phosphate (S1P). Treatment with TY52156, a selective antagonist for S1PR3, or genetic silencing using S1PR3-siRNA suppressed the viability of TSC2-deficient cells. Both SPHK1 and S1PR3 inhibitors markedly exhibited antitumor effect in a xenograft model of TSC2-null cells, restored autophagy level, and triggered cell death. Together, we identified novel rapamycin-insensitive sphingosine metabolic signatures in TSC2-null LAM cells. Therapeutic targeting of aberrant SPHK1/S1P/S1PR3 signaling may have potent therapeutic benefit for patients with TSC/LAM or other hyperactive mTOR neoplasms with autophagy inhibition.

## Introduction

Lymphangioleiomyomatosis (LAM) is a devastating pulmonary disease caused by mutation of tuberous sclerosis complex 1/2 (TSC1/2), resulting in mammalian target of rapamycin complex 1 (mTORC1) activation [1], which chiefly affects young women of childbearing age [2]. mTORC1 is activated by upregulated Rheb due to the mutations or defects of *TSC1* and *TSC2* and then promotes downstream phosphorylation of S6 and 4E-BP1 in LAM cells, contributing to increased protein synthesis to support growth and metabolism [3], meanwhile autophagy level is suppressed. According to clinical reports, the mTORC1 inhibitor rapamycin suppresses tumor growth and improves dyspnea symptoms in LAM, but lung function is declined after drug withdrawal [4, 5]. Therefore, exploration into mTORC1-independent signaling pathways regulated by TSC2 is urgently need to provide new targets for the treatment of LAM.

Recently, increasing evidence has investigated that sphingolipids play vital roles in the regulation of cancer cell growth and death [6]. S1P is a central molecule of sphingolipid metabolism that determines the fate of cells and is produced from sphingosine by two enzymes (SPHK1, SPHK2) [7]. S1P binding with five G-protein-coupled receptors (S1PR1-5s) regulates cell survival, migration and immune response [8, 9]. Growing evidence h.as shown that SPHK1 hyperactivation facilitates the development and progression of tumors [10]. In addition, researchers show that targeting the S1P axis attenuates inflammation and the formation of metastatic foci induced by obesity in breast cancer [11].

In this study, metabolic profiling identified the obviously abnormal upstream sphingolipid metabolites in TSC2-deficient cells. We revealed that TSC2 deficiency negatively regulated the expression of SPHK1 in a rapamycin-insensitive manner, which was a novel and previously unexplored finding in TSC/LAM research. Mechanistically, the higher expression of S1PR3 triggered tumorigenesis of TSC2-deficient cells by regulating cell viability, migration, and mediating an anti-apoptosis effect. Furthermore, we validated that inhibition of SPHK1 and S1PR3 triggered autophagic cell death independent of mTORC1 signaling and suppressed the growth of xenograft tumors in severe combined immunodeficient (SCID) mice models.

Considering the critical role of SPHK1/S1P/S1PR3-regulated autophagy in the progression of cancer [12, 13], we hypothesize that abnormal sphingolipid metabolism may be exploited as a novel therapeutic target in LAM treatment and that leveraging sphingolipid metabolism combined with mTORC1 inhibitor treatment may improve therapeutic strategies for LAM, TSC, and related diseases.

## Materials and methods

### Cell culture and reagents

LAM patient-derived 621-101 and 621-103 cells [14, 15], Eker rat uterine leiomyoma-derived (ELT3) cells [16], TSC2^−/−^p53^−/−^, TSC2^+/+^p53^−/−^ MEFs, and HEK293T cells were cultured in DMEM with 10% FBS, 1% penicillin/streptomycin. and HEK293T cells. All cultures were incubated at 37 °C in a humidified 5% CO_2_ atmosphere. Rapamycin (20 nM; ENZO), PF543 (2 μM; Cayman), TY52156 (2 μM; Cayman), W123 (10 μM; Cayman), Spingosine-1-phosphate (2 μM; Cayman), were used as indicated.

### Cell viability assay

Cells were seeded at a density of 8 × 10^4^ ml in 96-well plates and incubated for 24 h and then treated with inhibitors or vehicle at the indicated doses and durations. Cell viability was determined by MTT assay (Sigma). Values are expressed as the means ± SEM; *n* = 8/group.

### Cell death assay

Cells were plated in the 96-well black plates at a density of 8 × 10^4^ ml and treated with inhibitors or vehicle at the indicated doses and durations, with subsequent incubation with propidium iodide (5 μmol/L) in 100 μl PBS for 45 min at 37 °C. Fluorescence was read at 530 nm/ 620 nm. The data are represented as the percentage of dead cells relative to the total number of cells as detected by crystal violet staining. Values are expressed as the means ± SEM; *n* = 8/group.

### Transwell migration/invasion experiments

In all, 8–10 × 10^4^ cells per well were seeded in the upper chamber in 200 μl of serum-free medium or serum-free medium containing inhibitors, and 750 μl of 20% FBS medium was added to the lower chamber with or without matrigel. After incubation at 37 °C for 48 h, the cells remaining at the upper surface of the membrane removed with cotton swabs, and the cells on the lower surface of the membrane were considered to be migrated cells. The cells were fixed in 10% formalin for 10 min and then stained with 0.05% crystal violet for 10 min. The cells were examined, counted, and imaged under a microscope. The numbers of cells in five random fields of each chamber were counted and averaged.

### siRNA transfections

Human *S1PR3* siRNAs (50 nmol/L, Genepharma, #1, #2, #3) and human *TSC2* siRNAs (designed by Invitrogen) were transfected into cells using lipofectamine 2000 RNAIMAX (Invitrogen) according to the manufacturer’s protocols. Cells were harvested 48 h after transfection. The siRNA sequences were listed in the supplementary information.

### Stable cell line establishment

Short hair RNA (shRNA) used to silence SPHK1 (shSPHK1) or vector control was transfected into 293T cells together with auxiliary plasmids pLP1, pLP2, and VSV-G to package lentivirus. After infecting by the lentivirus, the cells selected by adding puromycin into the medium at a certain concentration. The shRNA was designed and synthesized by GenePharma (Shanghai, China).

### Immunoblotting and antibodies

Cells were lysed in RIPA buffer. All primary antibodies were diluted 1:1000 in 3% bovine serum albumin (BSA) dissolved in 1× TBST. Antibodies used were as follows: acid ceramidase (BD Biosciences, 612302), Tuberin/TSC2 (CST, #4308) Sphingosine phosphate kinase 1 (Abcam, ab71700), S1PR3 (Cayman, 10006373), S1PR1 (Cayman, 10005228), Phospho-62 (CST, #88588), Phospho-S6 (Ser235/236) (CST, #4858), cleaved-PARP (CST, #9548), cleaved-caspase 3 (CST, #9661), LC3B (CST, #3868), p44/42 MAPK (ERK1/2) (CST, #4696), p-AKT (CST, #9271), AKT (CST, #9272) and β-actin (Santa Cruz, sc-47778). All the original western blots are shown in Supplemental Material.

### H&E staining

The tumor tissue of mice was fixed with 4% paraformaldehyde for 2 days, embedded in paraffin and sectioned. After deparaffinization and gradient alcohol hydration, tissue paraffin sections (5 μM) were stained with hematoxylin–eosin (H&E) Staining Kit (Solarbio Life Science, Beijing, China). The numbers of cells in three random fields of each slide were imaged.

### Immunohistochemistry (IHC)

ASAH1 (BD Biosciences, 612302), SPHK1 (Abcam, ab71700), phosphor-S6 (CST, #4858) phospho-62 (CST, #88588) and Ki67 (CST, #9449) expression profiles in human colorectal cancer and normal tissues were analyzed by IHC. Paraffin-embedded tissue sections were dewaxed with xylene, re-hydrated with an ethanol gradient, and then incubated with fresh 3% H_2_O_2_ (diluted with methanol) for 10 min to eliminate endogenous peroxidase activity. The slides were blocked with 10% goat serum (diluted with PBS) at room temperature for 30 min and incubated with ASAH1, SPHK1, phospho-62, and Ki67 antibody (diluted 1:200 with 10% goat serum) at 4 °C overnight, followed by incubation with secondary antibody at room temperature for 30 min. Subsequently, the sections were stained with 3,3-diaminobenzidine (DAB) for 5–10 min and counterstained with hematoxylin. The numbers of cells in three random fields of each slide were imaged.

### TUNEL assay

A TUNEL assay was performed in deparaffinized 5-μm-thick sections using an Apoptosis Detection Kit (FITC) (Yeasen, 40306ES20) according to the supplier’s instructions. The sections were counterstained with hematoxylin. The numbers of cells in three random fields of each slide were imaged.

### Bioluminescent reporter imaging

Ten balb/c mice were randomized into two groups (*n* = 5, female, 18–20 g). For intravenous injections, 2 × 10^5^ 621-101shNC-Luc or 2 × 10^5^ 621-101 shSPHK1-Luc cells were injected into the lateral tail vein. Ten minutes before imaging, the animals were injected with luciferin (120 mg/kg; i.p.). Images were recorded using a Xenogen IVIS System. The total photon flux of the tumors in the chest regions and dissected lungs was analyzed.

### Gene expression analysis

Total RNA was isolated from cells with an RNAprep pure Cell/Bacteria Kit (TIANGEN, catalog No.#DP430), and 2 μg of RNA from each sample was reversed to cDNA with a First Strand cDNA Synthesis Kit (Thermo Scientific Fermentas, catalog No. 4368814). cDNA (0.5 μg) from each sample was used as a template to perform PCR, *n* = 6/group. The primer pairs for gene sequences are listed in Supplementary Information.

### Expression array analysis

Re-analysis of previously published expression array data (GEO accession no. GSE16944; [17]) was performed. The mRNA levels of sphingosine metabolism-related genes were compared between TSC2-deficient and TSC2-addback (TSC2 +) cells, or rapamycin- and vehicle-treated TSC2-deficient cells. The QC steps, normalization procedures and other detailed methodologies of microarray analysis are provided in the supplementary information.

### Animal studies

Luciferase-labeled ELT3-V3 cells (5 × 10^6^ cells in 100 μl of PBS) were injected subcutaneously into both flanks of SCID mice (female, 18–20 g). After ten days, the tumors were measured ~100 mm^3^ and the mice were randomly assigned to three groups according to the bioluminescence intensity (baseline): PF543 (2 mg/kg) group (*n* = 5) and TY52156 (2 mg/kg) group (*n* = 5), vehicle control group. Treatment was given to mice four times per week via intraperitoneal injection (i.p.), and the control mice was given vehicle. Tumor-bearing mice were sacrificed after 6 weeks of treatment. Tumor tissue was removed, and serum was collected.

### Statistical analysis

All data are shown as the means ± SEM of at least three independent experiments (*n* ≥ 3). The unpaired two-tailed Student’s *t* test was used to compare two groups, and two-way ANOVA was used for multiple-group comparisons. Statistical significance was defined as *P* < 0.05. No samples or animals were excluded from the analysis Graph production and statistical analysis were performed using GraphPad Prism (version 8.3.0).

## Results

### Identification of the upregulated sphingosine biosynthesis pathway in TSC2-deficient cells

**Fig. 1:**
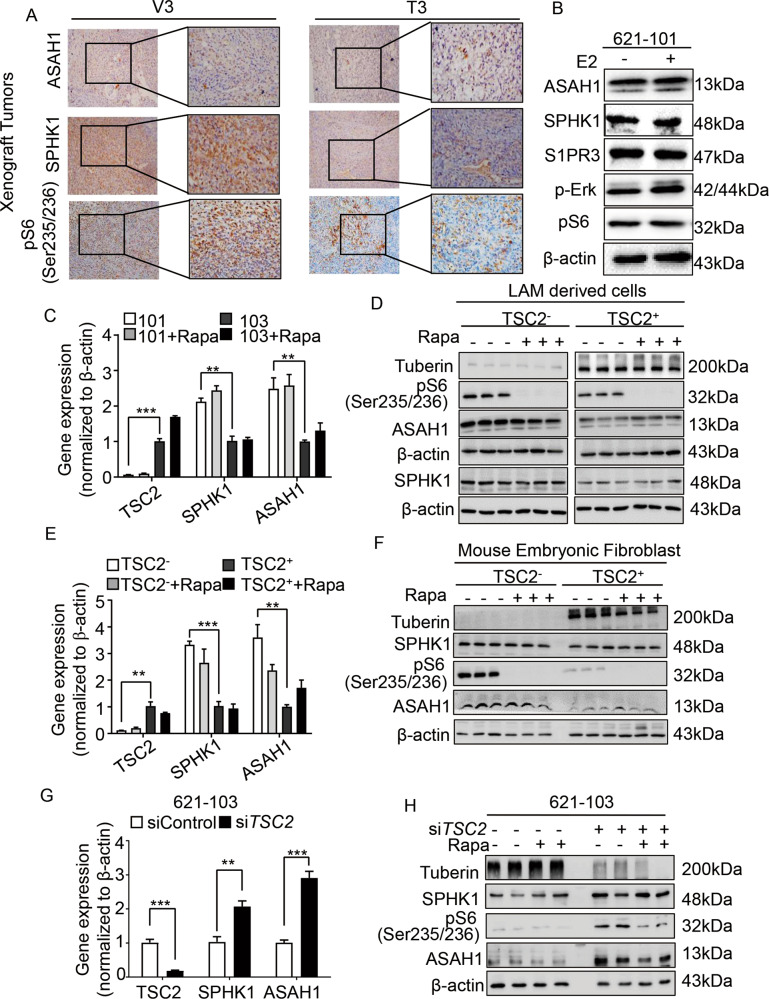
Identification of the upregulated sphingosine biosynthesis pathway in TSC2-deficient cells. **A** Immunohistochemistry staining of ASAH1, SPHK1 and phospho-S6(Ser235/236) was performed on rat-derived ELT3-V3 (TSC2-deficient, V3) and ELT3-T3 (TSC2-expression, T3) cell xenograft tumors of SCID mice. Representative images of five mice in each group. **B** Immunoblot analysis of ASAH1, SPHK1, S1PR3, p-ERK, pS6(235/236) protein levels in 621-101 cells treated with vehicle control or 10 nM estrogen for 24 h. β-actin was used as the loading control. **C**–**F** were treated with vehicle control or 20 nM rapamycin for 24 h. The mRNA levels of *TSC2, ASAH1*, and *SPHK1* were detected by RT-qPCR in (**C**) 621-101 and 621-103 cells, (**E**) Mouse embryo fibrosis cells (TSC2− MEFs and TSC2 + MEFs). Data showed the mean of three sets of independent samples. Protein expressions of tuberin, SPHK1, pS6(ser235/236) and ASAH1 were determined by immunoblot analysis in (**D**) 621-101 and 621-103 cells, (**F**) Mouse embryo fibrosis cells (TSC2^-^ MEFs and TSC2^+^ MEFs). β-actin was used as loading control. **G**, **H** TSC2-addback cells (621-103) were transfected with human *TSC2* siRNAs or control siRNA for 24 h. Gene expressions of *TSC2*, *SPHK1*, and *ASAH1* were determined by RT-qPCR, and protein levels of Tuberin, SPHK1, pS6(ser235/236) and ASAH1 were detected by immunoblotting. β-actin was used as loading control. Student’s *t* test, *P < 0.05, **P < 0.01, ***P < 0.001, ****P < 0.0001.

To examine the effects of sphingolipid in TSC2-deficient cells, we performed a metabolomics screen first and found a significant increase of ASAH1 and SPHK1 (Supplementary Fig. S1). Immunohistochemistry stain supported the findings that SPHK1 and ASAH1 exhibited the higher accumulation in tumors derived from ELT3-V3 cells and the overexpression of phosphate-S6 (ser235/236) confirmed that mTORC1 was activated (Fig. 1A and Supplementary Fig. S2). In contrast to the TSC2-addback cells, TSC2-deficient cells demonstrated a twofold increased SPHK1 expression at the mRNA and protein level (Fig. 1C, D). Previously, we reported female hormone estrogen facilitated COX2-mediated prostaglandin E2 biosynthesis and promoted the proliferation of TSC2-deficient cells [18]. So we treated TSC2-deficient cells with 10 nM estrogen to research the impact on sphingolipids. The results showed that the expressions of ASAH1, SPHK1, and S1PR3 were not changed (Fig. 1B and Supplementary Fig. S3).

Considering that hyperactive mTOR is a result of TSC2 deficiency, we detected the expression of SPHK1 treated with rapamycin in TSC2-deficient cells and TSC2-addback cells. The results did not show significant effects on either mRNA or protein level of SPHK1 but phosphate-S6 (ser235/236), downstream of mTORC1, was reduced, indicating that mTORC1 was not involved in the regulation of SPHK1 (Fig. 1C, D and Supplementary Fig. S4A). To confirm the relationship between TSC2 and sphingolipids, we tested the expression of SPHK1 and ASAH1 in other cell lines. Undoubtedly, rapamycin decreased the level of phosphate-S6 (ser235/236) but did not reduce the accumulation of SPHK1 and ASAH1 in TSC2^−/−^ p53^−/−^ Mouse embryo fibrosis cells (MEFs) and TSC2^+/+^ p53^−/−^ MEFs at the mRNA and protein level (Fig. 1E, F and Supplementary Fig. S4B), the similar results were also observed in ELT3 cell lines (Supplementary Fig. S5A–C). Besides, the results demonstrated that SPHK1 and ASAH1 were predominantly increased in *TSC2*-siRNA transfected 621-103 cells and HEK293T cells compared with the control-siRNA cells at the mRNA and protein level, and rapamycin did not impact them but suppressed phosphate-S6 (ser235/236) (Fig. 1G, H and Supplementary Figs. S6 and S7A–C). Collectively, these findings indicate that TSC2 negatively regulates the expression of SPHK1 in an mTORC1-independent manner.

### SPHK1 mediates tumorigenesis of TSC2-deficient cells in vitro and in vivo

**Fig. 2:**
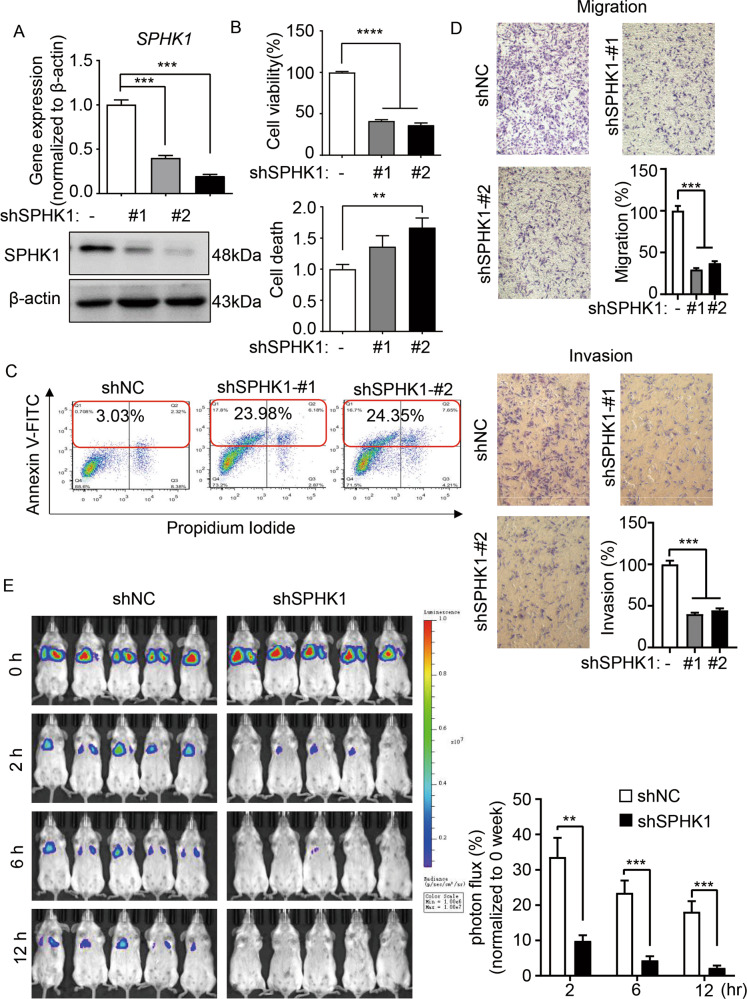
SPHK1 mediates tumorigenesis of TSC2-deficient cells in vitro and in vivo. **A** LAM patient-derived TSC2-deficient (621-101) cells were transfected with shRNA-*SPHK1* (#1 and #2) or a negative control (shNC). The mRNA and protein levels of SPHK1 were determined by RT-qPCR and immunoblotting, respectively. **B** Viability of 621-101shNC and 621-*101 shSPHK1* cells cultured in serum-free medium for 24 h was measured by MTT assay (upper panel). The cell death rate was measured by PI/CV assay (lower panel). The results are representative of eight independent samples per group. **C** 621-101shNC and 621-*101 shSPHK1* cells were seeded in 6-well plates for 24 h and then stained with an Annexin V:FITC apoptosis detection kit. Cell death was analyzed by flow cytometry. **D** The capacities for 621-101shNC and 621-*101 shSPHK1* cell migration (upper panel) and invasion (lower panel) were determined in a Transwell chamber without or with Matrigel. Cells were cultured in serum-free medium for 48 h and stained with crystal violet. Representative images of three repeats in each group. **E** 621-101shNC and 621-*101 shSPHK1* cells transfected with firefly luciferase were injected intravenously into SCID mice (*n* = 5). Lung colonization was determined by bioluminescence imaging 0 h, 2 h, 6 h, and 12 h post injection. Luminescence color scale: 0 h, 2 h, 6 h, and 12 h (min = 1 × 10^6^, max = 1 × 10^7^). The statistical analysis (lower panel) indicated the relative photon flux change. The results are representative of five mice per group. Student’s *t* test, **P < 0.01, ***P < 0.001, and ****P < 0.0001.

To evaluate the role of SPHK1 in the proliferation of TSC2-deficient cells, we knocked down *SPHK1* using two independent shRNA-*SPHK1* (sh*SPHK1*#1 and sh*SPHK1*#2). The mRNA and protein levels of SPHK1 were predominantly depleted relative to the negative control shRNA (shNC) (Fig. 2A and Supplementary Fig. S8). Moreover, the cell viability of sh*SPHK1* (#1 and #2) was reduced and the number of PI-positive cells was marked increased, indicating depleted SPHK1 resulted in decreased cell proliferation and elevated cell death (Fig. 2B). Next, we found that 621-101-sh*SPHK1* (#1 and #2) cells exhibited an increase of the population of Annexin V plus PI-positive cells, which were 23.98% and 24.35%, respectively, compared to 3.03% of the 621-101-shNC cells, indicating a critical role of SPHK1 in regulating apoptosis in TSC2-deficient cells (Fig. 2C). Further, the stained pictures and quantitative bar-charts showed that the migration and invasion of sh*SPHK1* (#1 and #2) cells were decreased compared with shNC cells (Fig. 2D). As reported, LAM cells spread via lymphatics and access to the lungs and exhibit a strong capacity to metastasize and colonize in remote tissues [19]. To investigate the impact of *SPHK1* depletion in regulating in vivo colonial ability of TSC2-deficient cells, 621-101-shNC and 621-101-sh*SPHK1* cells engineered with stable-luciferase expressing were inoculated intravenously into SCID mice. The noninvasive bioluminescence imaging indicated that within 30 min post injection (baseline), all mice in the two different groups exhibited the same level of bioluminescence intensity in the lungs. However, 2 h after injection, the bioluminescence of 621-101-sh*SPHK1* group was diminished threefold faster than that of 621-101-shNC. Then, at 6 h and 12 h, the bioluminescent signals of 621-101-sh*SPHK1* group continued to be quickly decreased compared with the shNC group, suggesting the effect of SPHK1 in sustaining lung colonization of TSC2-deficient cells in vivo (Fig. 2E).

### Inhibition of SPHK1 with PF543 decreases the survival of TSC2-deficient cells in vitro

**Fig. 3:**
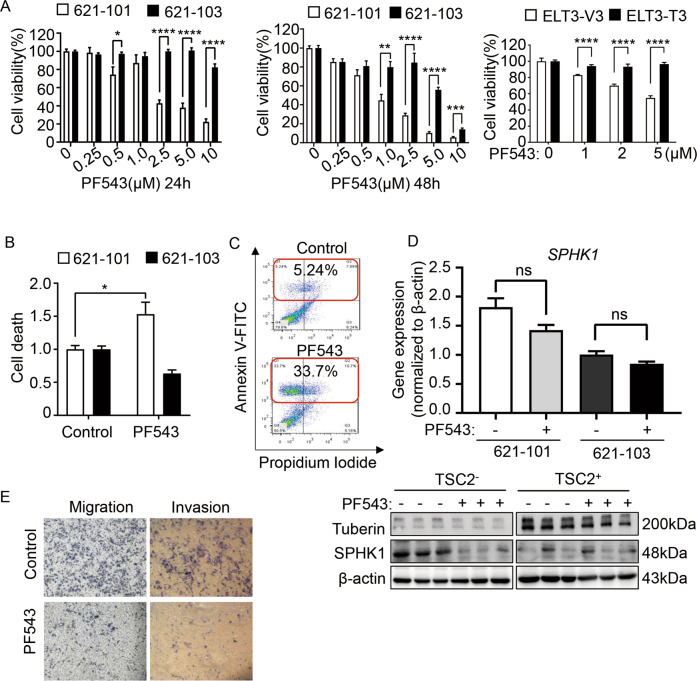
Inhibition of SPHK1 with PF543 decreases the survival of TSC2-deficient cells in vitro. **A** Cell viability of 621-101 and 621-103 cells treated with the specific SPHK1 inhibitor PF543 at the indicated concentrations for 24 h (left panel) and 48 h (middle panel) and ELT3-V3 and ELT3-T3 treated with the indicated concentrations of PF543 for 24 h (right panel) was measured by MTT assay in serum-free condition. The results are representative of eight independent samples per group. **B** The death of 621-101 and 621-103 cells treated with vehicle control or PF543 (2.5 μM) in serum-free conditions for 24 h was evaluated by PI/CV assay. The results are representative of eight independent samples per group. **C** 621-101 cells treated with vehicle control or PF543 (2.5 μM) for 24 h were stained with the reagent in an Annexin V: FITC apoptosis detection kit. Cell death was analyzed by flow cytometry (*n* = 3). **D** The mRNA and protein levels of SPHK1 in TSC2-deficient 621-10 cells and TSC2-addback 621-103 cells treated with vehicle control or PF543 were determined by RT-qPCR (upper panel) and immunoblot assay (lower panel). β-actin was used as the loading control. **E** Migration (left panel) and invasion (right panel) of 621-101 cells treated with vehicle control or PF543 (2.5 μM) under serum-free conditions for 48 h were measured in Transwell chambers without or with Matrigel. The cells were stained with crystal violet. Representative images of three repeats in each group. Student’s *t* test, *P < 0.05, **P < 0.01, ***P < 0.001, and ****P < 0.0001.

Next, we defined the role of SPHK1 in mediating tumorigenic function of TSC2-deficient cells using pharmacological compounds. We first treated TSC2-deficient and TSC2-addback cells with a potent SPHK1 inhibitor PF543, which showed higher affinity for SPHK1 than for SPHK2, in a concentration- and time-dependent manner. At concentrations from 2.5 µM to 10 µM, PF543 markedly decreased the viability of TSC2-deficient cells compared with TSC2-addback cells. The 50% efficiency of inhibition was obtained at a dose of 2.5 µM after 24 h. Treatment for 48 h, 1 µM PF543 suppressed the viability of TSC2-deficient cells by 50% but the TSC2-addback cells were negligibly affected. Notably, 10 µM PF543 led to complete suppression of cell viability in both TSC2-deficient and TSC2-addback cells, indicative of induced cytotoxicity. The viability of ELT3-V3 cells was also reduced by PF543. (Fig. 3A). Furthermore, PI exclusion staining showed a dramatic increase in TSC2-deficient cell-specific death, indicating that PF543 potently induced the death of TSC2-deficient cells (Fig. 3B). To further investigate how pharmacological inhibition of SPHK1 mediates the death of TSC2-deficient cells, we analyzed the apoptosis rate by flow cytometry. The number of Annexin V- and PI double-positive PF543-treated TSC2-deficient cells was not increased compared with that of the control cells, but the accumulation of Annexin V-positive cells showed that treatment with PF543 resulted in programmed cell death (Fig. 3C). Interestingly, we found that PF543-treated TSC2^+/−^ cells showed the unchanged mRNA level of SPHK1, but led to changes at the protein level (Fig. 3D and Supplementary Fig. S9). Moreover, we investigated that PF543 markedly suppressed the capacities of migration and invasion of TSC2-deficient cells (Fig. 3E). Collectively, our findings suggest that PF543 decreases the survival of TSC2-deficient cells.

### S1PR3 mediates S1P-promoted tumorigenesis of TSC2-deficient cells

**Fig. 4:**
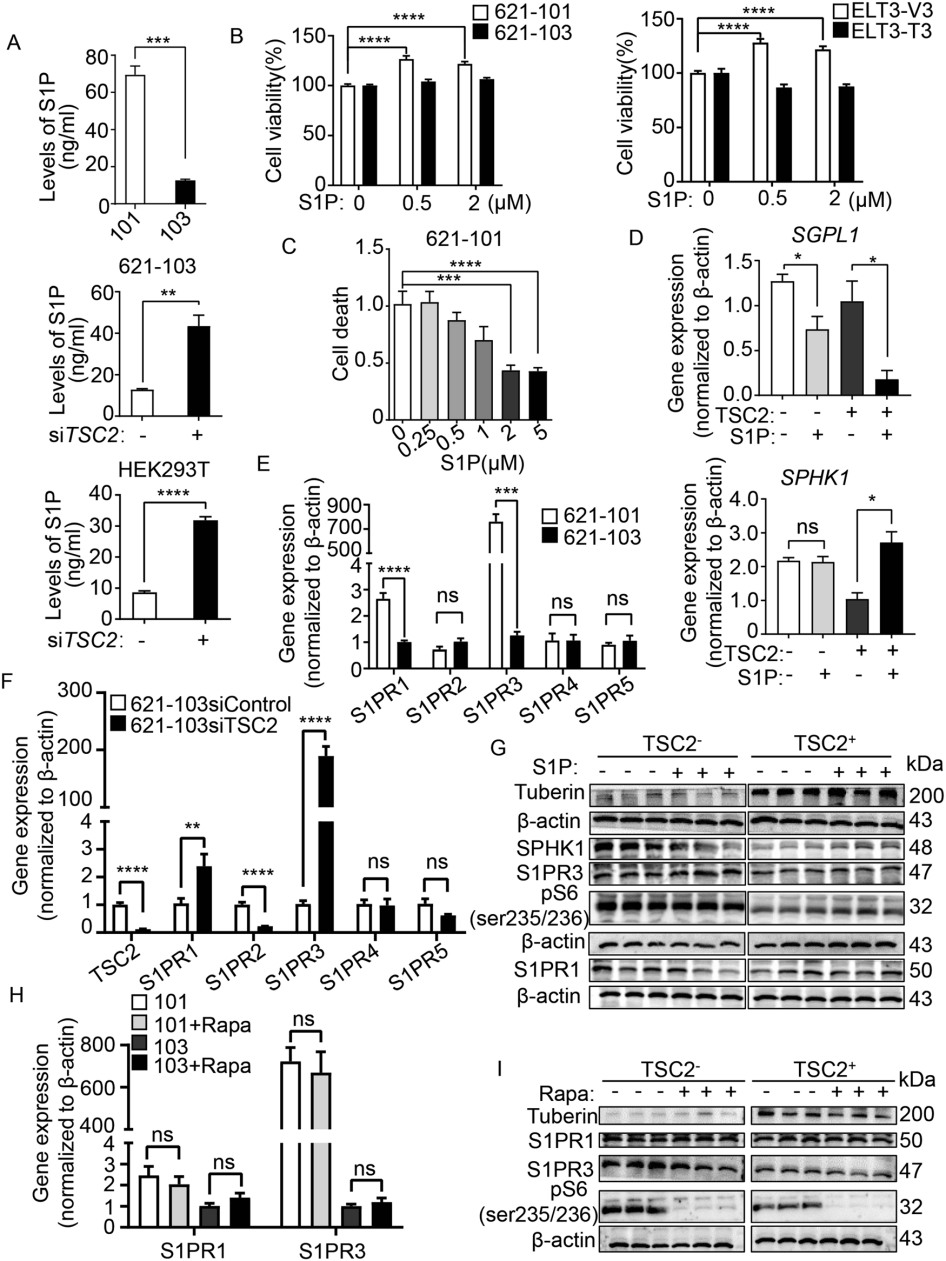
S1PR3 mediates S1P-promoted tumorigenesis of TSC2-deficient cells. **A** S1P in conditioned medium of cultured 621-101 and 621-103 cells (upper panel) or 621-103 cells (middle panel) and HEK293T cells (lower panel) transfected with siRNA-*TSC2* or siControl was quantified by ELISA. The Data were normalized to the control group. The results are representative of six sets of independent samples per group. **B** Viability of 621-101 and 621-103 cells (left panel) or ELT3-V3 and ELT3-T3 cells (right panel) treated with S1P for 24 h at the indicated concentrations was measured by MTT assay under serum-free conditions. **C** The death rate of 621-101 cells treated with 2 μM S1P for 24 h was determined by PI/CV assay under serum-free conditions. **B**, **C** Results are representative of eight independent samples per group. **D** RT-qPCR analyzed the mRNA level of SGPL1 and *SPHK1* in 621-101 and 621-103 cells treated with 2 μM S1P or vehicle control for 24 h. The data show the mean of three sets of independent samples. **E** The mRNA levels of *S1PR1*-*S1PR5* in 621-101 and 621-103 cells were detected by RT-qPCR assay. **F** RT-qPCR analyzed the mRNA levels of *TSC2*, *S1PR1*, *S1PR2*, *S1PR3*, *S1PR4*, and *S1PR5* in siRNA-*TSC2*-transfected 621-103 cells or siRNA-control cells. **G** 621-101 and 621-103 cells were treated with 2 μM S1P or vehicle control for 24 h. Tuberin, SPHK1, S1PR1, S1PR3, and phospho-S6 (S235/236) were analyzed by immunoblotting assay. β-actin was used as the loading control. The results are representative of three different experiments. **H**, **I** 621-101 and 621-103 cells were treated with 20 nM rapamycin or vehicle control for 24 h. The *S1PR1* and *S1PR3* levels were performed by RT-qPCR (**H**). Protein levels of tuberin, S1PR1, S1PR3, and phospho-S6 (S235/236) were determined by immunoblotting (**I**). β-actin was used as the loading control. The results are representative of three different experiments. Student’s *t* test, *P < 0.05, **P < 0.01, ***P < 0.001, and ****P < 0.0001.

SPHK1 catalyzes the conversion of sphingosine to S1P, which regulates numerous cellular processes and is involved in inflammation and cancer [20, 21]. To determine the impact of altered expression SPHK1 on sphingolipid metabolism, we quantified and found that the level of S1P was markedly higher in TSC2-deficient cells than that in TSC2-addback cells (Fig. 4A). In contrast, the sphingosine level was elevated in TSC2-addback cells, with lower expression of SPHK1 (data not shown).

To further define the interrelation of TSC2 and SPHK1, we knocked down *TSC2* in 621-103 cells using siRNA. In comparison to control siRNA, *TSC2*-siRNA cells exhibited a conspicuously increased level of S1P. Similar results were observed in HEK293T cells (Fig. 4A). To examine the biological effects of S1P in TSC2-deficient cells, we added exogenous S1P to evaluate their proliferative function. The results showed that S1P significantly promoted the viability of TSC2-deficient cells compared with TSC2-addback cells (Fig. 4B). Meanwhile, the number of cell death exhibited the opposite results, indicating the anti-death function of S1P (Fig. 4C). Two fates await S1P in cells: degradation by S1Plyse (encoded by the gene SGPL1) or by binding with receptors1–5 (S1PR1–5). SGPL1 supports lymphocyte trafficking and cancer process [22]. Next, we found that S1P decreased the mRNA level of SGPL1 but did not affect SPHK1 in TSC2-deficient cells, indicating that S1P exerted its function via S1PR1-5 (Fig. 4D). Interestingly, SGPL1 expression level was markedly reduced in TSC2-addback cells, possibly due to the lower level of substrate S1P, further confirming that SGPL1 was not involved in the core pathway in LAM cells. S1PRs regulate various cell functions, including cancer growth metastasis, and expresses in different tissues [23–26]. To identify the specific S1PRs in TSC2-deficient cells, we examined the differential expression of S1PRs. The mRNA levels of S1PR3 and S1PR1 were 700–800-fold and 2.5-fold higher, respectively, in TSC2-deficient cells than in TSC2-addback cells. However, the expressions of S1PR2, S1PR4 and S1PR5 did not change (Fig. 4E). Then, we discovered that the mRNA level of S1PR3 was increased in TSC2-siRNA transfection of TSC2-addback cells, but the other receptors either were decreased (S1PR1, S1PR2, S1PR5) or was not affected (S1PR4), indicating the crucial role of TSC2 in regulating S1PR3 (Fig. 4F). Accordingly, we found additional S1P supplement increased the protein level of S1PR3 in TSC2-deficient cells (Fig. 4G and Supplementary Fig. S10). To determine whether mTORC1 regulates the expressions of S1PR3, the TSC2-deficient cells were treated with rapamycin and the results showed that mTORC1 did not change the mRNA level of S1PR3 or S1PR1 (Fig. 4H). Next, we discovered that regardless of the absence or presence of TSC2, rapamycin did not alter the S1PR3 protein level but significantly reduced the expression of phosphate-S6 (ser235/236) in both TSC2-deficient and TSC2-addback cells (Fig. 4I and Supplementary Fig. S11). Collectively, these data indicate that S1PR3 plays a critical role in response to SPHK1, and TSC2 negatively regulates the expression of S1PR3 in a mTORC1-independent manner.

### Blocking S1PR3 decreases the survival of TSC2-deficient cells in vitro and in vivo

**Fig. 5:**
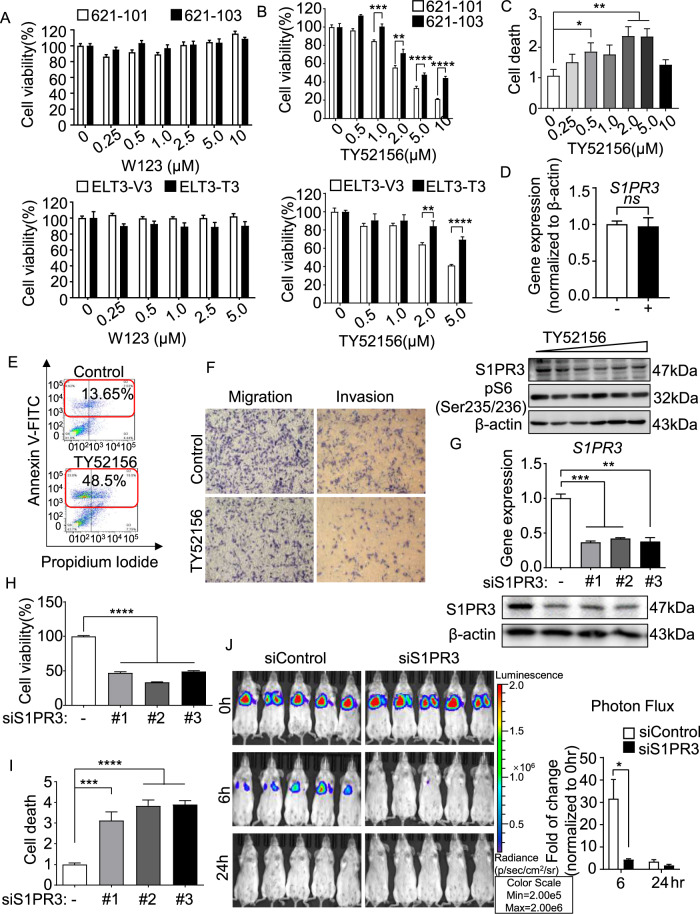
Blocking S1PR3 decreases the survival of TSC2-deficient cells in vitro and in vivo. **A**, **B** Viability of 621-101 and 621-103 cells (upper panel) and ELT3-V3 and ELT3-T3 cells (lower panel) treated with the S1PR1 inhibitor W123 (**A**) or S1PR3 inhibitor TY52156 (**B**) at the indicated concentrations for 24 h was measured by MTT assay. (**C**) The death rate of 621-101 and 621-101 cells treated with TY52156 at the indicated concentrations for 24 h was measured by PI/CV assay. **A**–**C** Results are representative of eight independent samples per group. **D** The mRNA level of *S1PR3* detected by RT-qPCR (upper panel) and the protein expression of S1PR3 and phosphor-S6 (235/236) detected by immunoblotting (lower panel) in vehicle control or TY52156-treated 621-101 cells were analyzed. **E** 621-101 cells treated with vehicle control or TY52156 (2 μM) were stained with Annexin V: FITC apoptosis detection kit reagent. Cell death was analyzed by flow cytometry (*n* = 3). **F** Migration (left panel) and invasion (right panel) of 621-101 cells treated with vehicle control or TY52156 (2 μM) were detected in Transwell chambers without or with Matrigel. **G** 621-101 cells were transfected with three independent *S1PR3* siRNAs (#1, #2, or #3) or the negative control (siControl). The mRNA and protein levels of S1PR3 were determined using RT-qPCR (upper panel) and immunoblotting (lower panel). **H**, **I** Viability (**H**) and death rate (**I**) of 621-101siControl and 621-101si*S1PR3* (#1, #2, or #3) cells were measured by MTT or PI/CV assay. The results are representative of eight independent samples per group. **J** 621-101siControl and 621-101si*S1PR3* cells stably expressing the luciferase tag were injected intravenously into SCID mice (*n* = 5). Lung colonization was quantified using bioluminescence intensity 0 h, 6 h, and 24 h post injection. Luminescence color scale: 0 h, 6 h, and 24 h (min = 2 × 10^5^, max = 2 × 10^6^). The statistical analysis (right panel) indicates the relative fold change in photon flux. The results are representative of five mice per group. Student’s *t* test, *P < 0.05, **P < 0.01, ***P < 0.001, and ****P < 0.0001.

To determine whether augmented S1PRs have any biological impact on cell survival, we treated cells with the potent antagonists that were specific for S1PRs. The S1PR1 inhibitor W123 did not affect the viability of TSC2-deficient cells, although S1PR1 had higher expression compared with TSC2-addback cells at the mRNA level (Fig. 5A). In contrast, TY52156, an S1PR3-specific antagonist, markedly decreased the viability of TSC2-deficient cells than TSC2-addback cells in a dose-dependent manner. Notably, 10 μmol/L TY52156 led to complete reduction in viability for 79% of TSC2-deficient cells, indicating cytotoxicity. Similar results were observed in ELT3-V3 cells (Fig. 5B). Moreover, TY52156 (2 µmol/L) induced a threefold increase in the number of dead TSC2-deficient cells (Fig. 5C). The results validated the inhibition effects of TY52156 in S1PR3 protein level in a dose-independent manner but not in mRNA level (Fig. 5D and Supplementary Fig. S12). To further analyze the apoptosis of TSC2-deficient cells induced by TY52156, the flow cytometry was used. A twofold increase of Annexin V and PI double stain cells and a fivefold accumulation of Annexin V-positive stain cells showed that treatment with TY52156 may result in programmed apoptosis (Fig. 5E). Next, the transwell assay found that TY52156 markedly weakened the capacities of migration and invasion of TSC2-deficient cells, indicating that inhibition of S1PR3 reduced the metastatic capacity of tumor cells (Fig. 5F).

To define the role of genetic S1PR3 depletion in the viability of TSC2-deficient cells, we knocked down S1PR3 using siRNA transfection. siRNA-S1PR3 (siS1PR3#1–3), relative to negative control siRNA (siControl), markedly decreased the protein and mRNA levels of S1PR3 (Fig. 5G and Supplementary Fig. S13). Moreover, siS1PR3#1 exhibited a threefold increase of cell death and significantly reduced cell viability by 53% compared with siControl cells. The siS1PR3#2 and siS1PR3#3 results were consistent with before (Fig. 5H, I). Next, we established a lung colonization model to validate the effect of S1PR3 in TSC2-deficient cells. Six hours after cells were intravenously injected into mice, the reduction of fluorescence intensity in the siS1PR3 group was faster than that in the siControl group (Fig. 5J). Collectively, our findings demonstrate that S1PR3 is a critical regulator of the survival of TSC2-deficient cells.

### Inhibition of SPHK1/S1PR3 signaling triggers autophagic death in TSC2-deficient cells

**Fig. 6:**
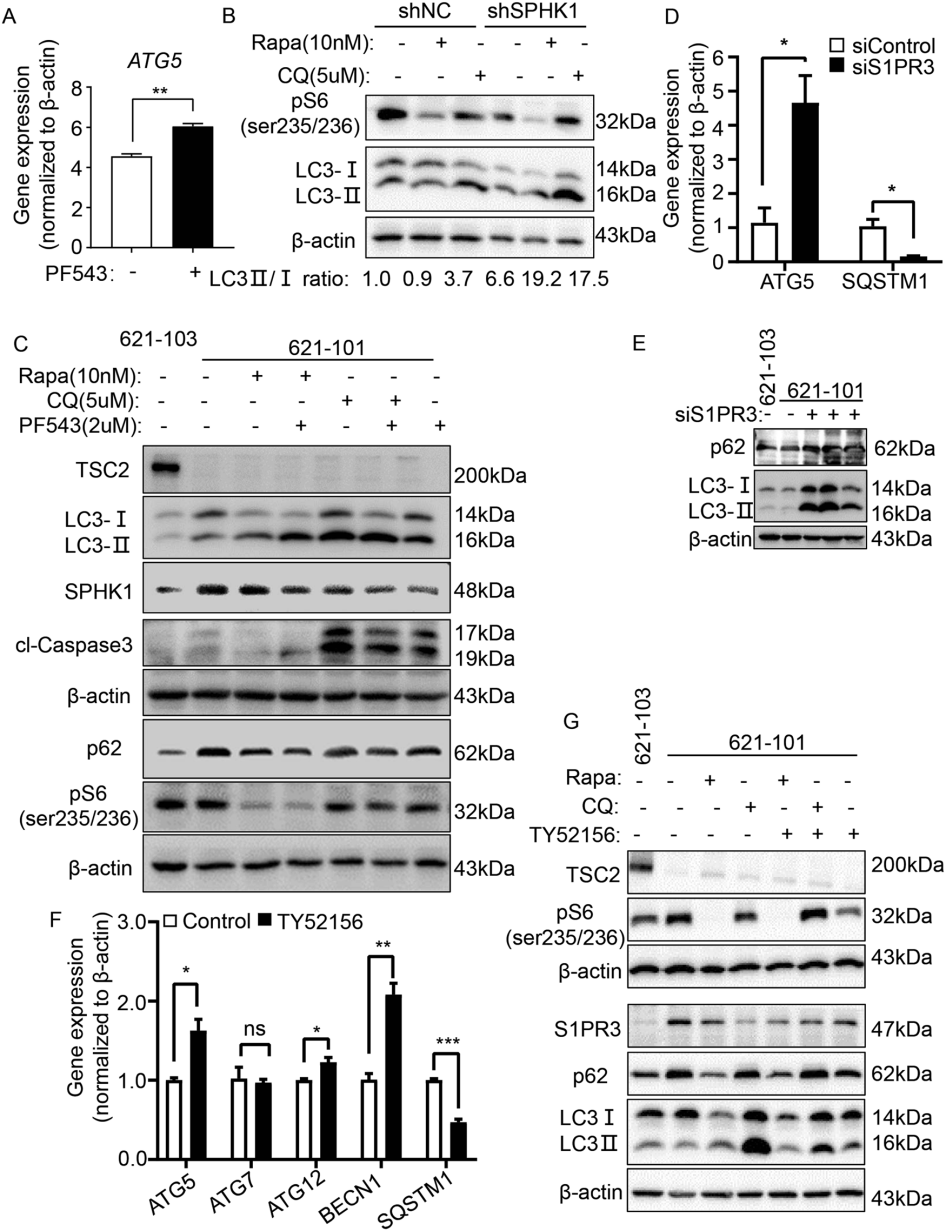
Inhibition of SPHK1/S1PR3 signaling triggers autophagic death in TSC2-deficient cells. **A** Gene expression of *ATG5* in 621-101 cells treated with vehicle control or specific SPHK1 inhibitor PF543 (2.5 μM) was detected by RT-qPCR. 621-101 cells. **B** 621-101 cells were transfected with shRNA-*SPHK1* or negative control (shN.C). Immunoblotting was performed to determine the levels of phospho-S6 (235/236), LC3I and LC3II in 621-101 shRNA-*SPHK1* cells and control cells treated with vehicle control, rapamycin (Rapa, 10 nM) or CQ (5 µM). **C** 621-101 cells were treated with 20 nM rapamycin and 5 µM CQ, with or without 2.5 µM PF543 for 24 h. Levels of tuberin, p62, phospho-S6 (235/236), LC3I/LC3II, cl-caspase 3 and SPHK1 were assessed by immunoblotting. 621-103 cells were added as TSC2-addback control. **D**, **E** 621-101 cells were transfected with *S1PR3*-siRNA or negative control (siControl). Gene expression of *ATG5* and *SQSTM1* was analyzed by RT-qPCR (**D**), and protein levels of p62, LC3I/LC3II were determined by immunoblotting (**E**). **F** 621-101 cells were treated with vehicle control or selective S1PR3 inhibitor TY52156 (2 μM), and the gene expression of *ATG5*, *ATG7*, *ATG12*, *BECN1* and *SQSTM1* was detected by RT-qPCR. **G** 621-101 cells were treated with 20 nM Rapa and 5 µM CQ with or without 2 µM TY52156 for 24 h. Protein levels of tuberin, p62, phospho-S6 (235/236), LC3I/LC3II and S1PR3 were assessed by immunoblotting. 621-103 cells were added as TSC2-addback control. Student’s *t* test, *P < 0.05, **P < 0.01, ***P < 0.001.

To determine whether S1P signaling promotes cell survival by interacting with the mTORC1/autophagy pathway, the mRNA levels of autophagy-related genes were measured and the results indicated that they were not affected after depleting SPHK1 (Supplementary Fig. S14A). However, PF543 elevated autophagy-related gene 5 (ATG5) level in TSC2-deficient cells (Fig. 6A).

As previously reported, cell death was induced through bulk processes, including apoptosis and autophagy. We speculated that SPHK1-regulated S1P may be correlated with autophagy. So we measured the expression of the autophagy marker p62 and microtubule-associated protein light chain 3 (LC3II/LC3I) in SPHK1-depleted cells treated with the autophagy regulators rapamycin or chloroquine (CQ). As expected, rapamycin showed only a limited regulatory effect on autophagy in TSC2-deficient cells; however, with SHPK1 depletion, autophagy was dramatically elevated upon rapamycin stimulation, indicating that SPHK1 signaling may regulate autophagy independent of mTORC1 signaling (Fig. 6B).

Next, we treated both TSC2-deficient and TSC2-addback cells with rapamycin, CQ and PF543. PF543 showed an increased expression of LC3II in 621-101 cells. Interestingly, we discovered that PF543 triggered autophagy in 621-101 cells by reducing the accumulation of p62 and increasing the LC3II/LC3I ratio, regardless of whether rapamycin or chloroquine was used. Similar results were observed in ELT3-V3 cells (Fig. 6C and Supplementary Figs. S14B, S14C, S15). Together, these data imply that PF543 decreases the survival of TSC2-deficient cells via autophagy upregulation.

Similarly, S1PR3 was shown to participate in autophagy regulation. Depletion of S1PR3 induced the mRNA level of ATG5 and elevated the accumulation of LC3II in TSC2-deficient cells (Fig. 6D, E and Supplementary Fig. S16). Also, TY52156 significantly increased the levels of autophagy-related genes and upregulated the protein expression of LC3II (Fig. 6F, G and Supplementary Fig. S17). Interestingly, we studied that p-AKT/AKT and p-ERK/ERK were not changed after treatment of PF543 or TY52156 in TSC2-deficient cells (Supplementary Fig. S18A–D), revealed us that the cell fate regulated by SPHK1 and S1PR3 may be a relation with other signal pathways.

### Targeting SPHK1 and S1PR3 with inhibitors suppresses the growth of TSC2-deficient cell xenograft tumors

**Fig. 7:**
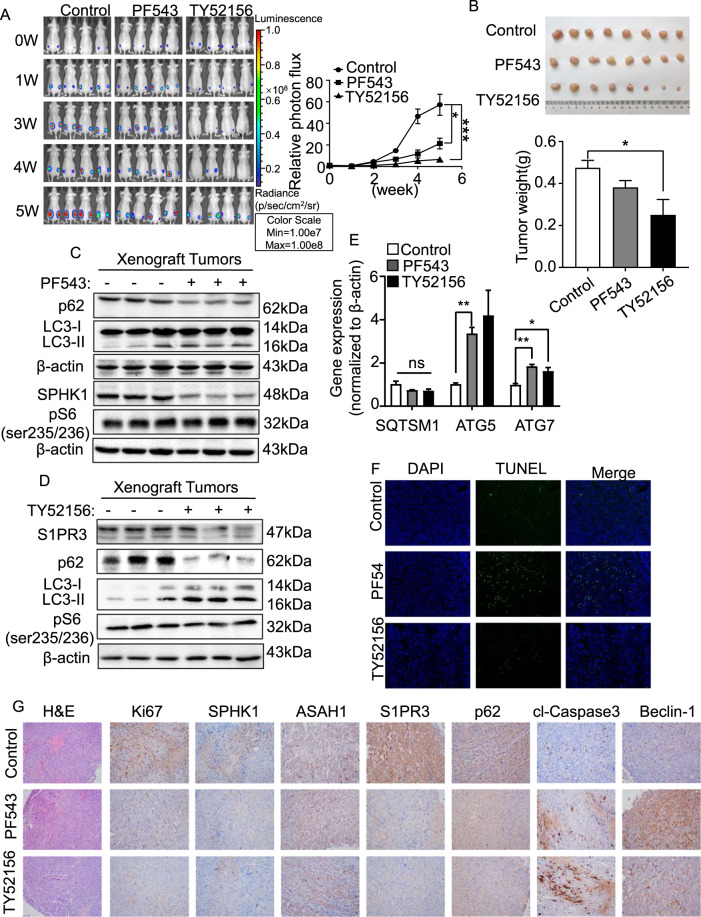
Targeting SPHK1 and S1PR3 with inhibitors suppress growth of TSC2-deficient cell xenograft tumors. Female SCID mice were inoculated with ELT3-V3 cells (stably expressing luciferase tag) subcutaneously. Tumor-bearing mice were intraperitoneally administered vehicle control, PF543 (2 mg/kg, four times per week) or TY52156 (2 mg/kg, four times per week). **A** Animals were given D-luciferin (200 μL per 20 g body weight, *i.p*.) before bioluminescence imaging was performed at the indicated times. Luminescence color scale: 0 week-5 week (min = 1 × 10^7^, max = 1 × 10^8^). The statistical analysis in the right panel indicates the photon flux. Relative photon flux was measured using Perkin Elmer software. The results are representative of five mice per group. **B** The size of subcutaneous tumors was measured after 5 weeks of treatment (upper panel), and the tumor weights were quantified (lower panel). **C**, **D** Immunoblot analysis of SPHK1 or S1PR3, phosphor-62, LC3I/LC3II and phosphor-S6 (Ser235/236) in xenograft tumors treated with vehicle control, PF543 or TY52156 was performed. Three tumors were randomly selected in each group. **E** Gene expression of *SQTSM1*, *ATG5* and *ATG7* in the xenograft tumors was detected by RT-PCR. The results represent tumors from five mice per group. **F** Analysis of the apoptosis rate was performed using a TUNEL kit with xenograft tumors treated with vehicle control, PF543 or TY52156. **G** H&E and IHC staining of SPHK1, S1PR3, ASAH1, p62, cl-caspase 3, beclin-1, and Ki67 in xenograft tumors of ELT3-V3 cells treated with vehicle control, PF543, or TY52156. The results represent tumors from five mice in each group. Student’s *t* test, *P < 0.05 and **P < 0.01.

To investigate the role of SPHK1 and S1PR3 in mediating the survival of TSC2-deficient cells in vivo, a xenograft tumor model was established using ELT3-V3-luciferase-expressing cells. After 5 weeks of treatment, the photon flux in the xenograft tumors was elevated 21% in the PF543 group and 6% in the TY52156 group relative to the baseline, but that in the control group was increased 57% (Fig. 7A), indicating that inhibition of SPHK1 and S1PR3 suppressed the growth of tumors in vivo. The same results were obtained through a tumor size analysis (Fig. 7B). The decreased protein levels of SPHK1 and S1PR3 were obtained in PF543 and TY52156 group, respectively. Interestingly, the protein level of phosphate-S6 (ser235/236) was not changed, suggesting that treatment with S1P signaling antagonists did not affect mTORC1 pathway. As we speculated, the protein level of p62 was reduced in PF543 and TY52156 group, and an increased LC3II/LC3I ratio was observed (Fig. 7C, D and Supplementary Fig. S19A, S19B). The mRNA level of ATG5 exhibited a significant accumulation in the two treated groups (Fig. 7E). These data suggest that PF543 and TY52156 trigger the progression of autophagy in TSC2-deficient cell xenograft tumors. To explore whether PF543 or TY52156 induces apoptosis in vivo, a TUNEL assay was performed. The results revealed that PF543 and TY52156 accelerated the apoptosis rate in the ELT3 xenograft tumor model (Fig. 7F). To demonstrate the effects of PF543 and TY52156, tumor tissues were stained with H&E and IHC. Representative images showed that the treatment group underwent a relatively complete morphology change, and the levels of Ki67, SPHK1, and S1PR3 were reduced. Furthermore, the protein expression of phospho-62 was significantly decreased in both treatment groups (Fig. 7G). Collectively, these data illustrate that inhibition of SPHK1 or S1PR3 triggers autophagic death in tumor tissues and slows the occurrence and development of tumors.

## Discussion

LAM develops from *TSC1* and *TSC2* mutations in women and these mutations lead to the functional loss of *TSC2* and hyperactivation of the mTORC1 pathway, promoting cell proliferation and metastasis [27]. Currently, the only clinical drug for LAM is rapamycin, which affects mTORC1 activation [28]. However, tumors can relapse after drug withdrawal because of immune suppression and other side effects [29, 30]. Therefore, effective therapeutic targets and LAM treatment methods need to be sought.

SPHK1, which produces S1P, seems to play a core role in cancer progression [31], although its characteristics in LAM are not clear. In this study, we identified the negative regulation of SPHK1 in TSC2-deficient cells (Fig. 1). Knocking down SPHK1 decreased the viability and migration of TSC2-deficient cells without affected TSC2-addback cells, and 621-101 shSPHK1-expressing luciferase cells exhibited weak capacity of lung colonization in vivo (Fig. 2). These results are in line with the research that siRNA-*SPHK1* results in an increased apoptosis, impaired proliferation, migration and invasion in neuroblastoma cells [32]. PF543 is a potent specific inhibitor of SPHK1, Cheresh et al find that PF543 mitigates pulmonary fibrosis and attenuates S1P production and mitochondrial DNA (mtDNA) damage in mouse lungs and cells [33]. Our results also suggested that PF543 reduced cell viability, weakened the migration and invasion, and increased the death of TSC2-deficient cells (Fig. 3). Interestingly, flow cytometry showed that PF543 principally induced Annexin V-positive cells but only slightly increased Annexin V and PI double-positive cells, revealing that the cell death induced by PF543 may be realized through other mechanisms (Fig. 3). Next, we found that PF543 promoted the expression of ATG5 and the ratio of LC3II/LC3I, and attenuated the protein level of phospho-62, indicating that the inhibited autophagy was restored and triggered cell death in TSC2-deficient cells (Fig. 6). It has been reported that sphingolipids impact autophagy by regulating the assembly of the autophagy machinery, and change the fusion between autophagosomes and lysosomes [34]. However, another research reveals that phosphatidic acid (PA), metabolized by diacylglycerol kinase alpha (DGKA), are increased in TSC2-deficient MEFs and a DGKA inhibitor ritanserin synergizes with CQ selectively inhibits the proliferation of TSC2-deficient cells, independently of autophagy [35]. It reminds us that the relationship between autophagy and PF53 in TSC-deficient cells needs to be further explored.

SPHK1 is the primary enzyme for S1P synthesis, and S1P has been confirmed to contribute to physiological and pathological process in cancer cells [36]. In our results, overexpression of SPHK1 led to accumulation of S1P in TSC2-deficient cells, and knockdown of TSC2 resulted in an identical increase in both 621-103 and HEK293T cells (Fig. 4). Next, we found that the intracellular activity of S1P is mainly associated with the S1PR3 in TSC2-deficient cells. Interestingly, the elevated level of S1PR3 was not affected by rapamycin (Fig. 4). TY52156, a potent inhibitor of S1PR3, suppressed the cell viability and decreased the migration ability of TSC2-deficient cells (Fig. 5). Shen et al. investigate that TY52156 inhibits the proliferation of osteosarcoma cells and facilitates their apoptosis in vitro [37]. In our studies, targeting the SPHK1/S1PR3 axis with inhibitors reduced the expression of SPHK1 and S1PR3 and the biosynthesis of S1P (data now shown), significantly suppressed TSC2-deficient cells metastasis and prolonged the survival of mice carrying xenograft tumor (Fig. 7). In this case, suppression of tumor survival showed a benefit from TY52156, but the effect of PF543 was not as dramatic as inhibiting S1PR3. The reason may be that S1P acts as a second messenger in cells, while S1PR3 is more directly involved in cell fate. Feng et al. demonstrate that lysophosphatidic acid receptor 1 (LPAR1) and S1PR3 are significantly overexpressed in TSC2-deficient cells and lysophospholipase D autotaxin (ATX) inhibitor, GLPG1690, inhibits the phosphorylation of AKT and Erk1/2 in TSC2-deficient cells [38]. Nevertheless, in this study, PF543 and TY52156 did not affect the expression of p-AKT and p-Erk1/2, indicating their effectiveness worked through other signaling pathways. These findings are in agreement with the role of the SPHK1/S1P/S1PR3 axis in enhancing aldehyde dehydrogenase-positive cancer-stem cells and subsequent Notch activation [39].

**Fig. 8:**
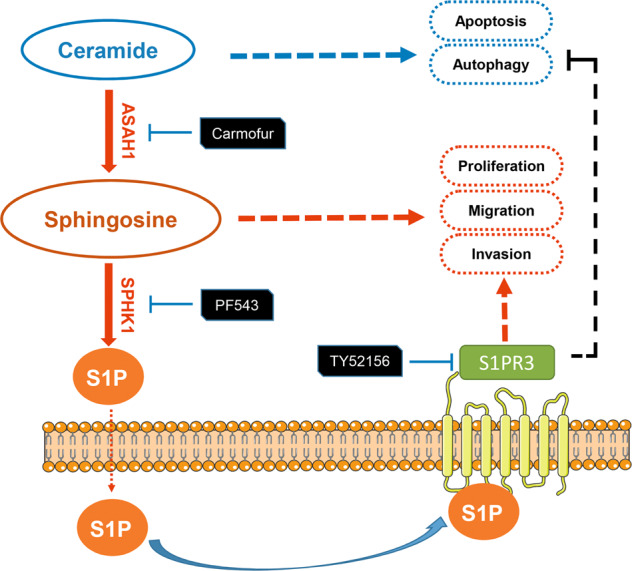
Schematic diagram of molecular mechanism. Simplified graph representation of abnormal sphingosine metabolism signaling that promotes tumorigenesis of TSC2-deficient cells.

Generally, our study elucidates the abnormal SPHK1/S1P/S1PR3 axis in LAM progression and the effects of PF543 and TY52156 in antitumor therapy (Fig. 8). Further studies of more specific compounds and exploration into combinations of clinical drugs that targeting SPHK1 and S1PR3 can offer aid in explaining their mechanism and exploring the treatment possibilities in LAM and other TSC2-related diseases.

## Supplementary information

Supplementary Figure

Supplementary Information

Supplemental material (WB)

Author contributions

aj-checklist

## Data Availability

All data generated or analyzed during this study are included in this published article and are available from the corresponding author upon reasonable request.
