# Supplementary material for: Targeting SPHK1/S1PR3-regulated S-1-P metabolic disorder triggers autophagic cell death in pulmonary lymphangiomyomatosis (LAM)
Source: Cell Death Dis. 2022 Dec 21;13(12):1065. doi: 10.1038/s41419-022-05511-3 (PMC9772321; doi:10.1038/s41419-022-05511-3)
Supplement: Supplementary file 1 — Supplementary Figure [file 41419_2022_5511_MOESM1_ESM.pptx]

## Slide 1
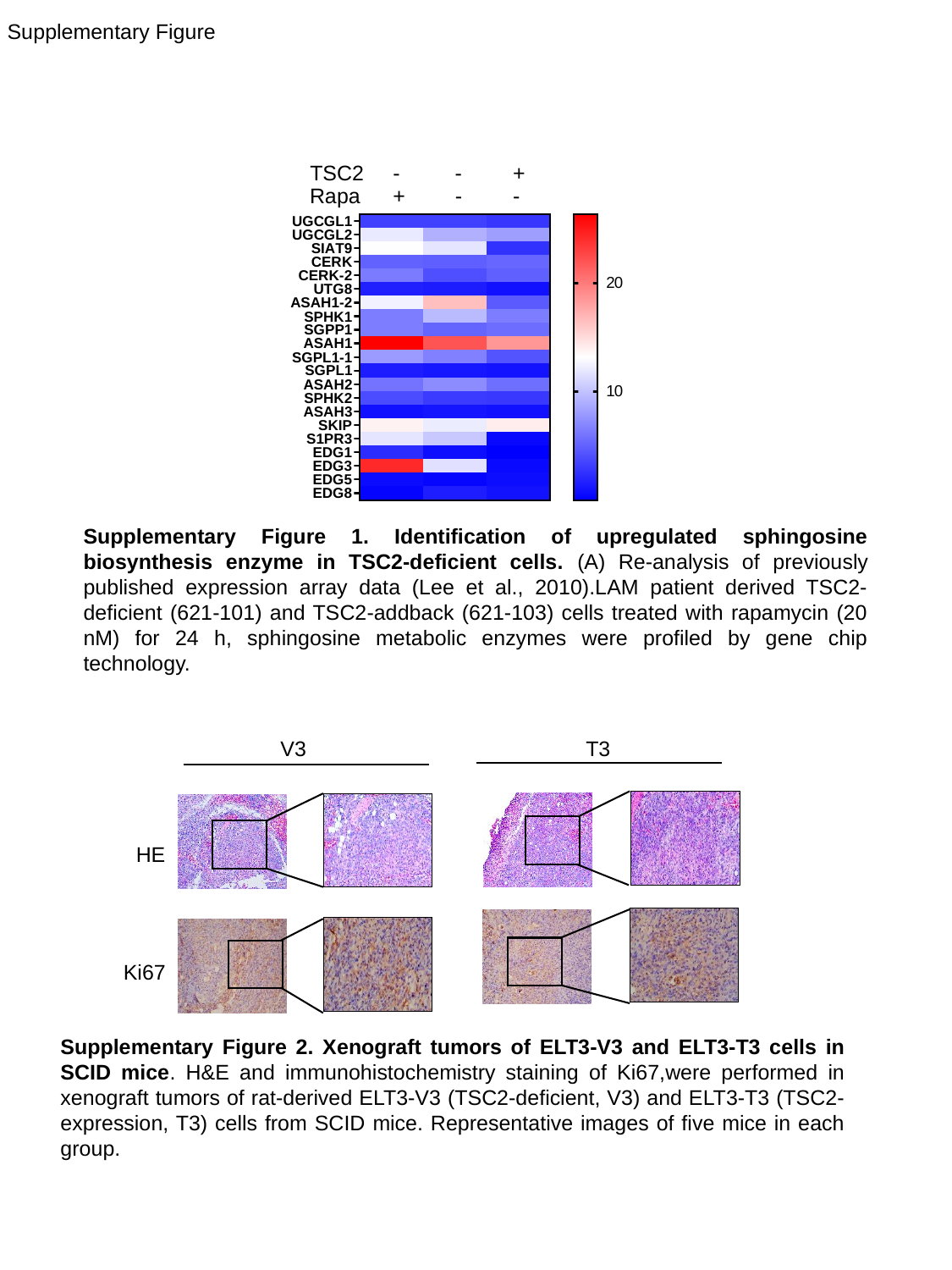

Supplementary Figure
-
TSC2
-
+
-
-
Rapa
+
Supplementary Figure 1. Identification of upregulated sphingosine biosynthesis enzyme in TSC2-deficient cells. (A) Re-analysis of previously published expression array data (Lee et al., 2010).LAM patient derived TSC2-deficient (621-101) and TSC2-addback (621-103) cells treated with rapamycin (20 nM) for 24 h, sphingosine metabolic enzymes were profiled by gene chip technology.
T3
V3
HE
Ki67
Supplementary Figure 2. Xenograft tumors of ELT3-V3 and ELT3-T3 cells in SCID mice. H&E and immunohistochemistry staining of Ki67,were performed in xenograft tumors of rat-derived ELT3-V3 (TSC2-deficient, V3) and ELT3-T3 (TSC2-expression, T3) cells from SCID mice. Representative images of five mice in each group.

## Slide 2
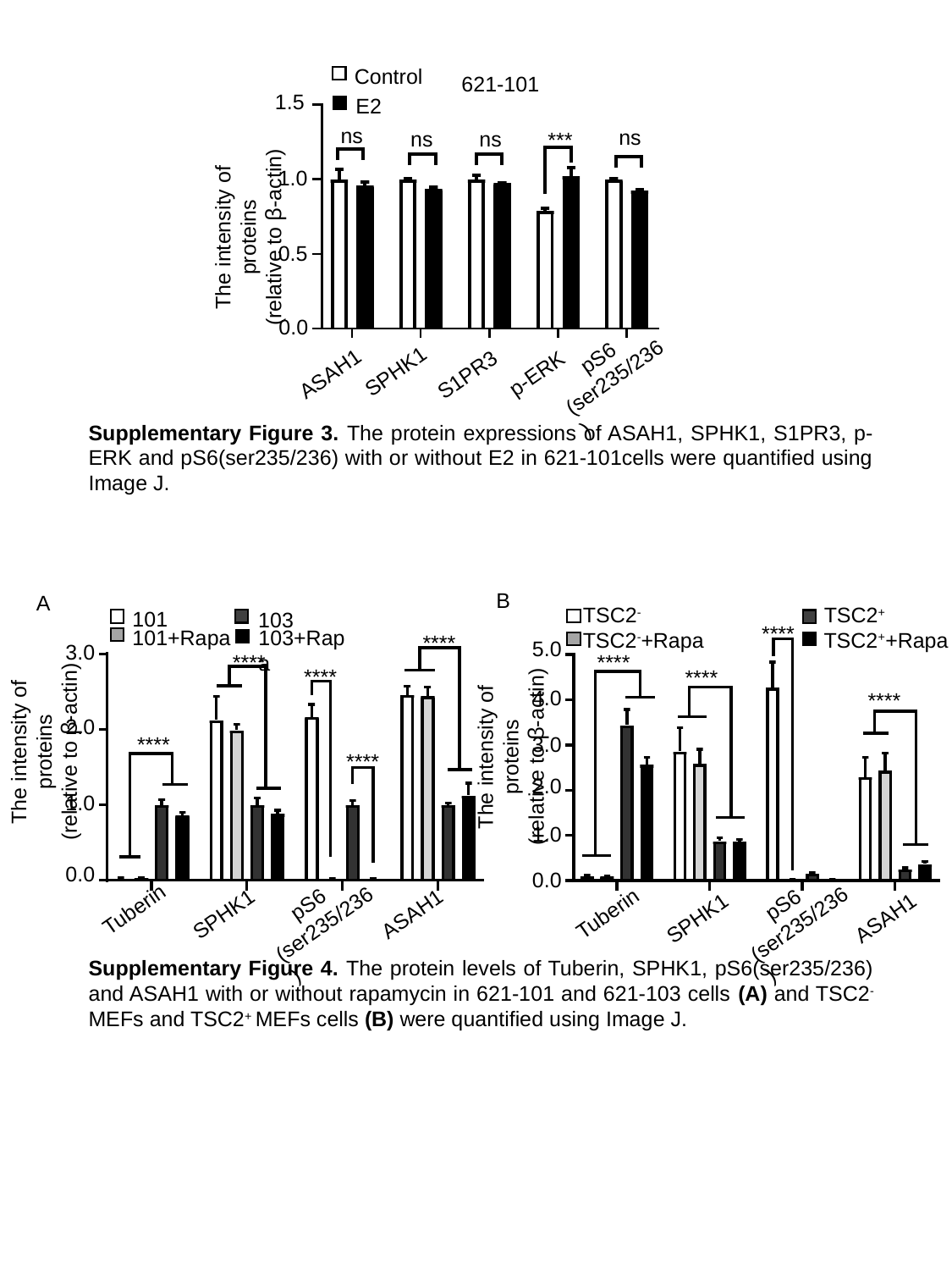

Control
621-101
1.5
The intensity of proteins
(relative to ꞵ-actin)
ns
ns
ns
ns
***
1.0
0.5
0.0
 pS6
(ser235/236)
p-ERK
SPHK1
ASAH1
S1PR3
E2
Supplementary Figure 3. The protein expressions of ASAH1, SPHK1, S1PR3, p-ERK and pS6(ser235/236) with or without E2 in 621-101cells were quantified using Image J.
B
A
TSC2-
TSC2+
****
TSC2++Rapa
TSC2-+Rapa
5.0
The intensity of proteins
(relative to ꞵ-actin)
****
****
4.0
****
2.0
0.0
 pS6
(ser235/236)
Tuberin
ASAH1
SPHK1
3.0
1.0
101
103
103+Rapa
101+Rapa
****
The intensity of proteins
(relative to ꞵ-actin)
3.0
****
****
2.0
****
 ****
1.0
0.0
 pS6
(ser235/236)
Tuberin
ASAH1
SPHK1
Supplementary Figure 4. The protein levels of Tuberin, SPHK1, pS6(ser235/236) and ASAH1 with or without rapamycin in 621-101 and 621-103 cells (A) and TSC2- MEFs and TSC2+ MEFs cells (B) were quantified using Image J.

## Slide 3
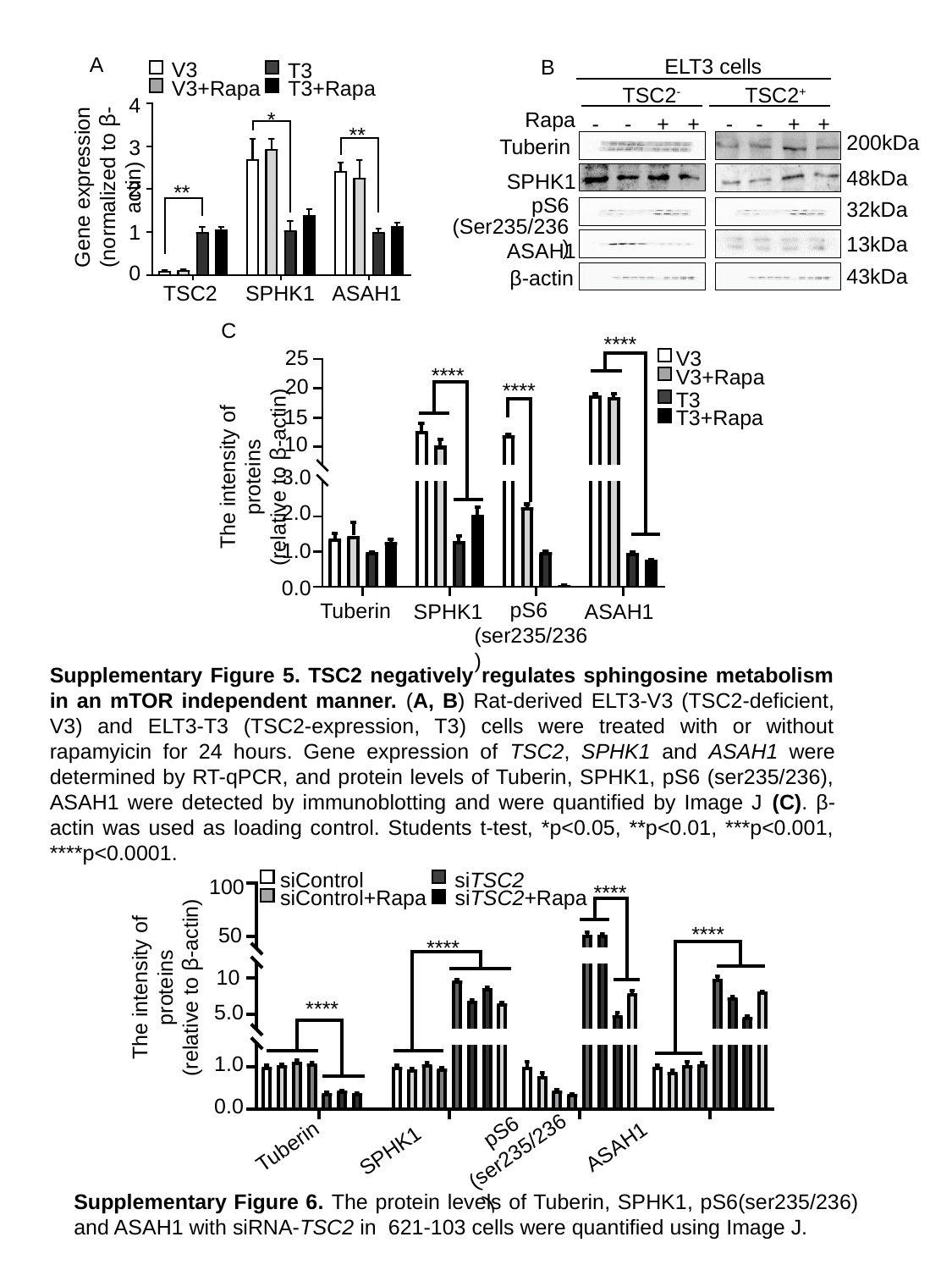

A
V3
V3+Rapa
T3
T3+Rapa
Gene expression
(normalized to ꞵ-actin)
 *
 **
**
TSC2
SPHK1
ASAH1
4
3
2
1
0
ELT3 cells
TSC2+
TSC2-
-
-
+
+
+
-
-
+
Rapa
Tuberin
SPHK1
ASAH1
β-actin
pS6
(Ser235/236)
B
200kDa
48kDa
32kDa
13kDa
43kDa
C
****
20
****
15
10
2.0
1.0
0.0
 pS6
(ser235/236)
Tuberin
SPHK1
ASAH1
V3
****
V3+Rapa
T3
T3+Rapa
The intensity of proteins
(relative to ꞵ-actin)
25
3.0
Supplementary Figure 5. TSC2 negatively regulates sphingosine metabolism in an mTOR independent manner. (A, B) Rat-derived ELT3-V3 (TSC2-deficient, V3) and ELT3-T3 (TSC2-expression, T3) cells were treated with or without rapamyicin for 24 hours. Gene expression of TSC2, SPHK1 and ASAH1 were determined by RT-qPCR, and protein levels of Tuberin, SPHK1, pS6 (ser235/236), ASAH1 were detected by immunoblotting and were quantified by Image J (C). β-actin was used as loading control. Students t-test, *p<0.05, **p<0.01, ***p<0.001, ****p<0.0001.
siControl
siTSC2
The intensity of proteins
(relative to ꞵ-actin)
****
****
50
****
10
****
5.0
1.0
0.0
 pS6
(ser235/236)
ASAH1
Tuberin
SPHK1
siControl+Rapa
siTSC2+Rapa
100
Supplementary Figure 6. The protein levels of Tuberin, SPHK1, pS6(ser235/236) and ASAH1 with siRNA-TSC2 in 621-103 cells were quantified using Image J.

## Slide 4
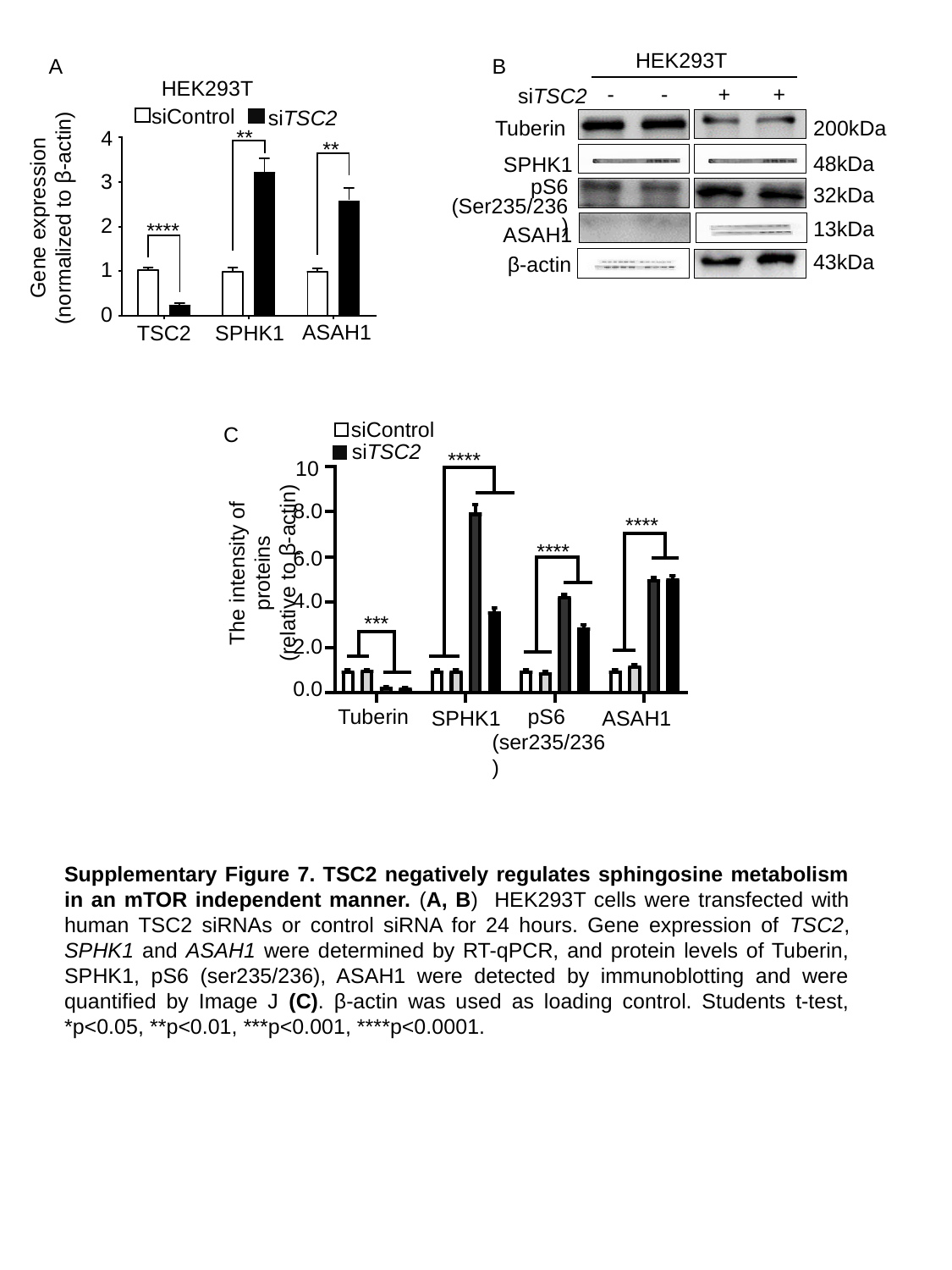

HEK293T
-
-
+
+
siTSC2
Tuberin
SPHK1
pS6
(Ser235/236)
ASAH1
β-actin
B
200kDa
48kDa
32kDa
13kDa
43kDa
A
HEK293T
Gene expression
(normalized to ꞵ-actin)
siControl
siTSC2
**
4
**
3
2
****
1
ASAH1
TSC2
SPHK1
0
siControl
siTSC2
****
 10
The intensity of proteins
(relative to ꞵ-actin)
****
****
***
 pS6
(ser235/236)
Tuberin
SPHK1
ASAH1
8.0
6.0
4.0
2.0
0.0
C
Supplementary Figure 7. TSC2 negatively regulates sphingosine metabolism in an mTOR independent manner. (A, B) HEK293T cells were transfected with human TSC2 siRNAs or control siRNA for 24 hours. Gene expression of TSC2, SPHK1 and ASAH1 were determined by RT-qPCR, and protein levels of Tuberin, SPHK1, pS6 (ser235/236), ASAH1 were detected by immunoblotting and were quantified by Image J (C). β-actin was used as loading control. Students t-test, *p<0.05, **p<0.01, ***p<0.001, ****p<0.0001.

## Slide 5
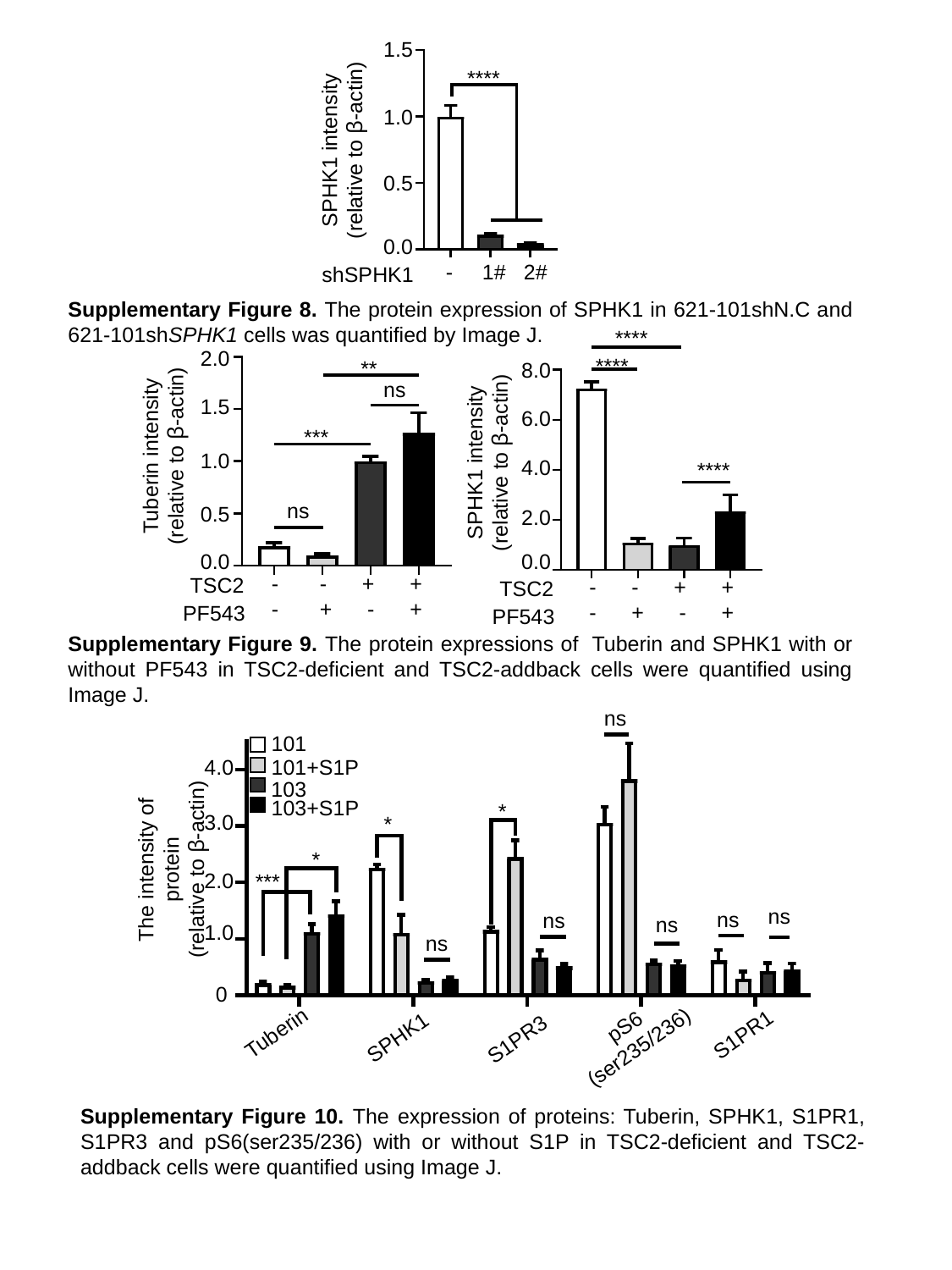

1.5
SPHK1 intensity
(relative to ꞵ-actin)
****
1.0
0.5
0.0
- 1# 2#
shSPHK1
Supplementary Figure 8. The protein expression of SPHK1 in 621-101shN.C and 621-101shSPHK1 cells was quantified by Image J.
****
****
SPHK1 intensity
(relative to ꞵ-actin)
6.0
4.0
****
2.0
0.0
 - - + +
 - + - +
TSC2
PF543
8.0
2.0
Tuberin intensity
(relative to ꞵ-actin)
 **
ns
1.5
***
1.0
ns
0.5
0.0
 - - + +
 - + - +
TSC2
PF543
Supplementary Figure 9. The protein expressions of Tuberin and SPHK1 with or without PF543 in TSC2-deficient and TSC2-addback cells were quantified using Image J.
ns
*
3.0
*
*
2.0
***
ns
ns
1.0
ns
0
 pS6
(ser235/236)
Tuberin
S1PR1
SPHK1
S1PR3
The intensity of protein
(relative to ꞵ-actin)
101
101+S1P
103
103+S1P
ns
4.0
ns
Supplementary Figure 10. The expression of proteins: Tuberin, SPHK1, S1PR1, S1PR3 and pS6(ser235/236) with or without S1P in TSC2-deficient and TSC2-addback cells were quantified using Image J.

## Slide 6
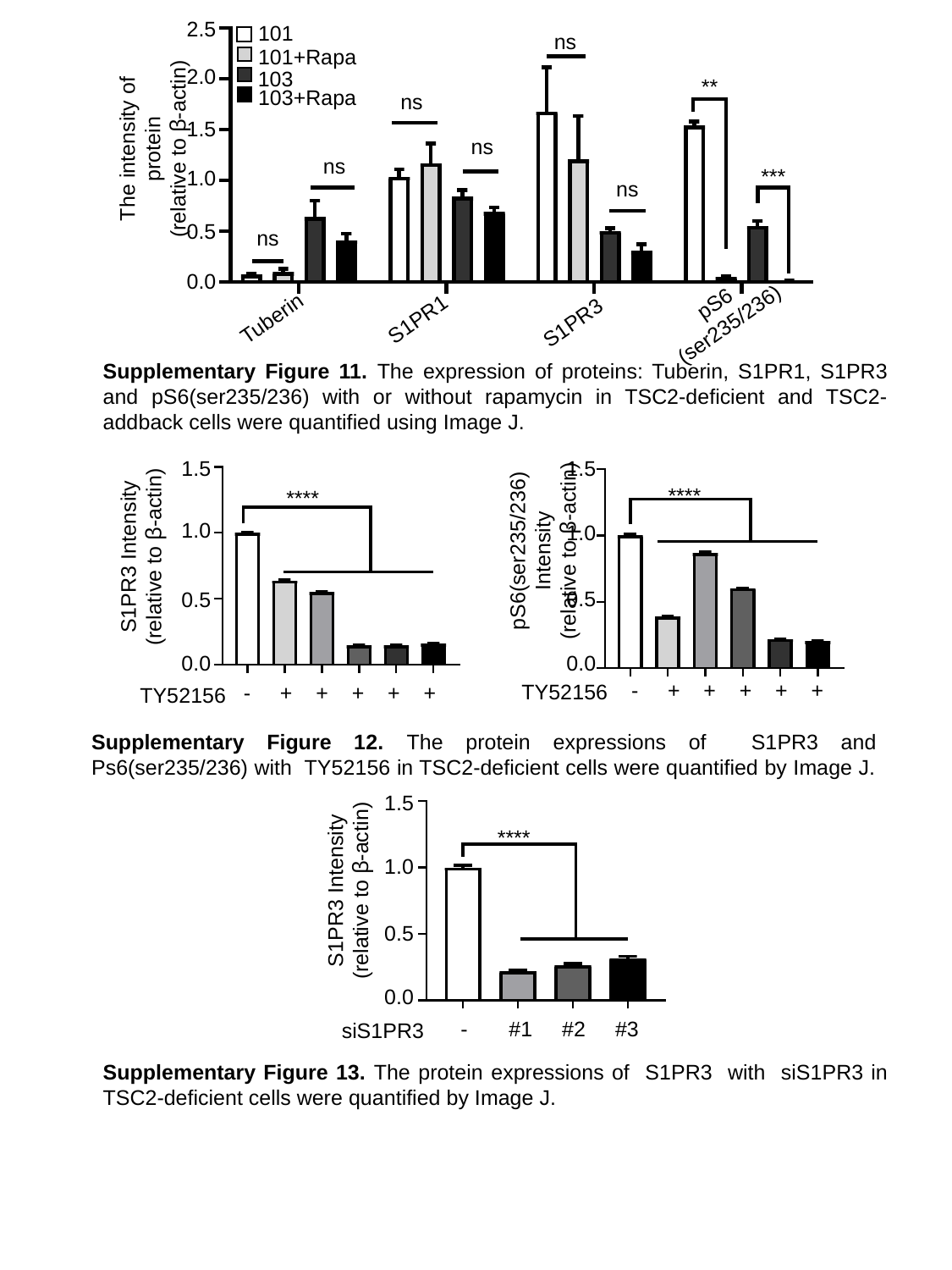

2.5
101
ns
The intensity of protein
(relative to ꞵ-actin)
101+Rapa
2.0
103
**
103+Rapa
ns
ns
ns
***
1.0
ns
ns
 pS6
(ser235/236)
S1PR1
Tuberin
S1PR3
1.5
0.5
0.0
Supplementary Figure 11. The expression of proteins: Tuberin, S1PR1, S1PR3 and pS6(ser235/236) with or without rapamycin in TSC2-deficient and TSC2-addback cells were quantified using Image J.
pS6(ser235/236) Intensity
(relative to ꞵ-actin)
1.5
****
1.0
0.5
0.0
 - + + + + +
TY52156
S1PR3 Intensity
(relative to ꞵ-actin)
1.5
****
1.0
0.5
0.0
 - + + + + +
TY52156
Supplementary Figure 12. The protein expressions of S1PR3 and Ps6(ser235/236) with TY52156 in TSC2-deficient cells were quantified by Image J.
S1PR3 Intensity
(relative to ꞵ-actin)
1.5
****
1.0
0.5
0.0
 - #1 #2 #3
siS1PR3
Supplementary Figure 13. The protein expressions of S1PR3 with siS1PR3 in TSC2-deficient cells were quantified by Image J.

## Slide 7
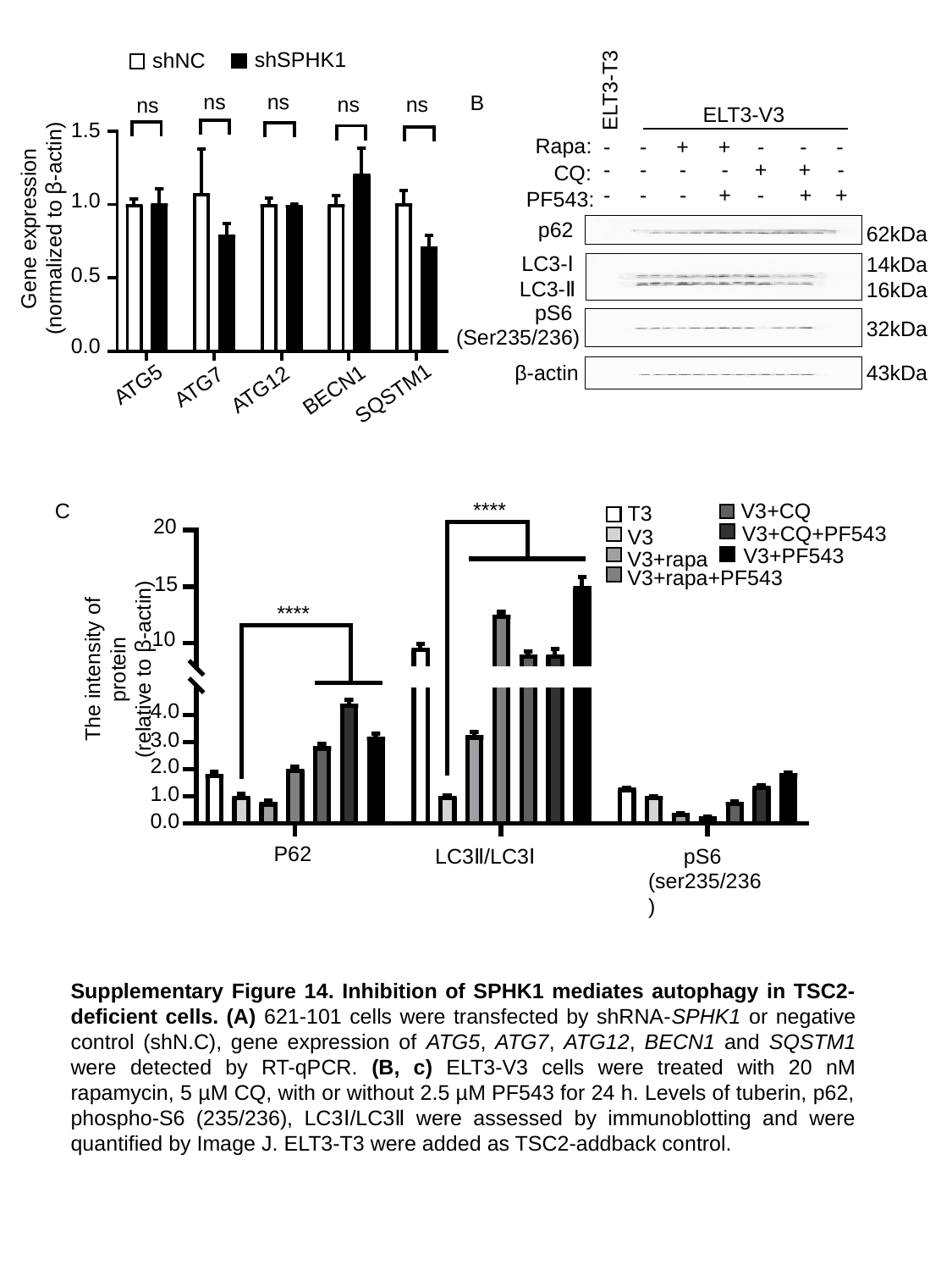

ELT3-T3
B
ELT3-V3
Rapa:
- - + + - - -
- - - - + + -
CQ:
- - - + - + +
PF543:
p62
LC3-Ⅰ
LC3-Ⅱ
 pS6
(Ser235/236)
β-actin
shSPHK1
shNC
1.5
1.0
0.5
0.0
Gene expression
(normalized to ꞵ-actin)
ATG5
ATG7
ATG12
BECN1
SQSTM1
ns
ns
ns
ns
ns
62kDa
14kDa
16kDa
32kDa
43kDa
****
V3+CQ
T3
20
V3+CQ+PF543
V3
V3+PF543
V3+rapa
The intensity of protein
(relative to ꞵ-actin)
V3+rapa+PF543
15
****
10
4.0
2.0
0.0
P62
 pS6
(ser235/236)
LC3Ⅱ/LC3Ⅰ
3.0
1.0
C
Supplementary Figure 14. Inhibition of SPHK1 mediates autophagy in TSC2-deficient cells. (A) 621-101 cells were transfected by shRNA-SPHK1 or negative control (shN.C), gene expression of ATG5, ATG7, ATG12, BECN1 and SQSTM1 were detected by RT-qPCR. (B, c) ELT3-V3 cells were treated with 20 nM rapamycin, 5 µM CQ, with or without 2.5 µM PF543 for 24 h. Levels of tuberin, p62, phospho-S6 (235/236), LC3Ⅰ/LC3Ⅱ were assessed by immunoblotting and were quantified by Image J. ELT3-T3 were added as TSC2-addback control.

## Slide 8
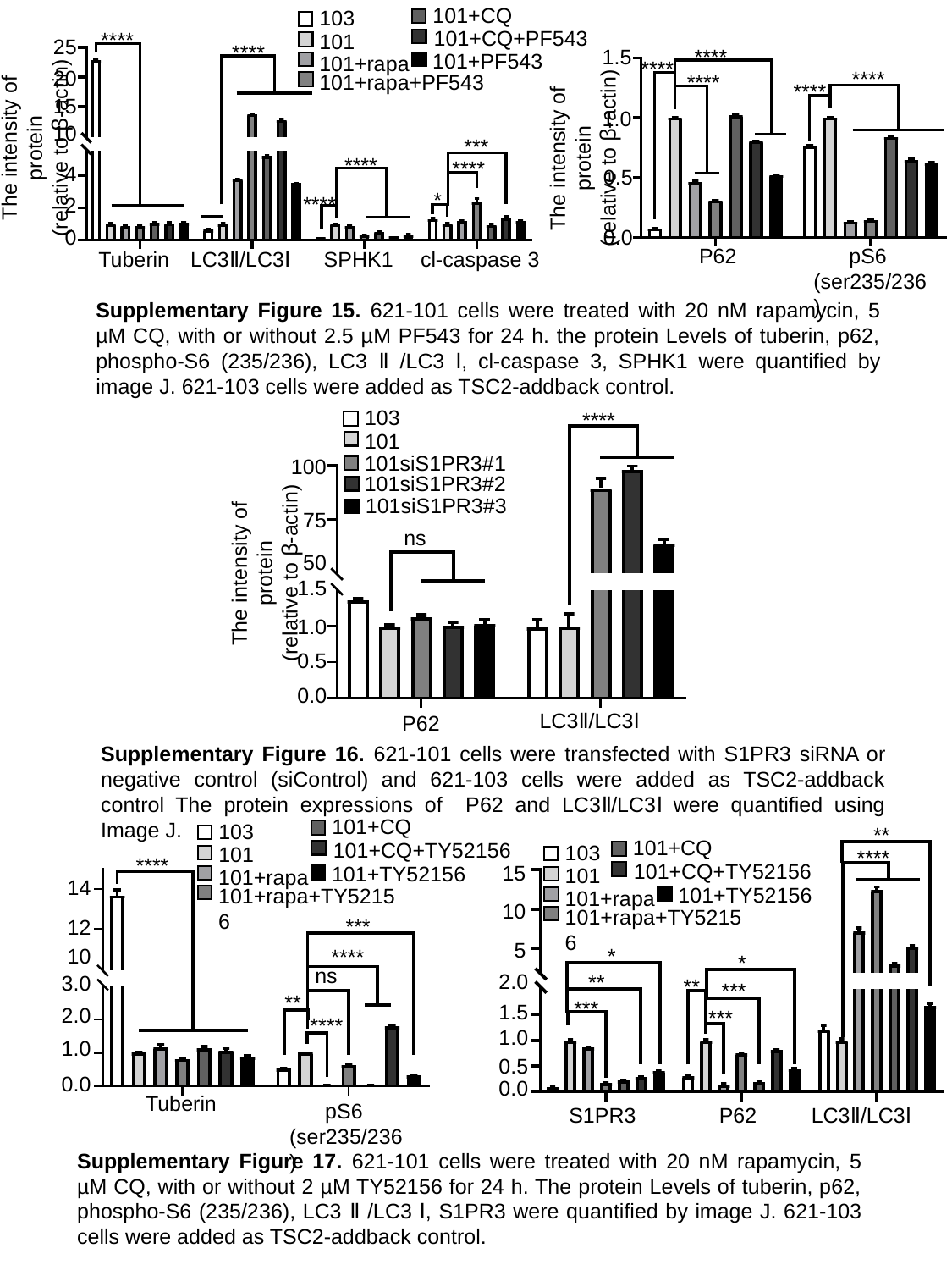

101+CQ
103
101+CQ+PF543
Tuberin
LC3Ⅱ/LC3Ⅰ
SPHK1
cl-caspase 3
****
25
The intensity of protein
(relative to ꞵ-actin)
****
20
15
10
***
****
****
4
*
****
2
0
101
101+PF543
101+rapa
101+rapa+PF543
****
1.5
The intensity of protein
(relative to ꞵ-actin)
****
****
****
****
1.0
0.5
0.0
P62
 pS6
(ser235/236)
Supplementary Figure 15. 621-101 cells were treated with 20 nM rapamycin, 5 µM CQ, with or without 2.5 µM PF543 for 24 h. the protein Levels of tuberin, p62, phospho-S6 (235/236), LC3 Ⅱ /LC3 Ⅰ, cl-caspase 3, SPHK1 were quantified by image J. 621-103 cells were added as TSC2-addback control.
103
****
101
101siS1PR3#1
100
The intensity of protein
(relative to ꞵ-actin)
101siS1PR3#2
101siS1PR3#3
75
50
1.5
1.0
0.5
0.0
LC3Ⅱ/LC3Ⅰ
P62
ns
Supplementary Figure 16. 621-101 cells were transfected with S1PR3 siRNA or negative control (siControl) and 621-103 cells were added as TSC2-addback control The protein expressions of P62 and LC3Ⅱ/LC3Ⅰ were quantified using Image J.
101+CQ
103
 **
****
15
10
 5
 *
 *
2.0
 **
 **
 ***
 ***
1.5
 ***
1.0
0.5
0.0
S1PR3
P62
LC3Ⅱ/LC3Ⅰ
101+CQ
103
101+CQ+TY52156
101
101+TY52156
101+rapa
101+rapa+TY52156
101+CQ+TY52156
101
****
14
***
12
10
****
 ns
3.0
 **
2.0
****
1.0
0.0
Tuberin
 pS6
(ser235/236)
101+TY52156
101+rapa
101+rapa+TY52156
Supplementary Figure 17. 621-101 cells were treated with 20 nM rapamycin, 5 µM CQ, with or without 2 µM TY52156 for 24 h. The protein Levels of tuberin, p62, phospho-S6 (235/236), LC3 Ⅱ /LC3 Ⅰ, S1PR3 were quantified by image J. 621-103 cells were added as TSC2-addback control.

## Slide 9
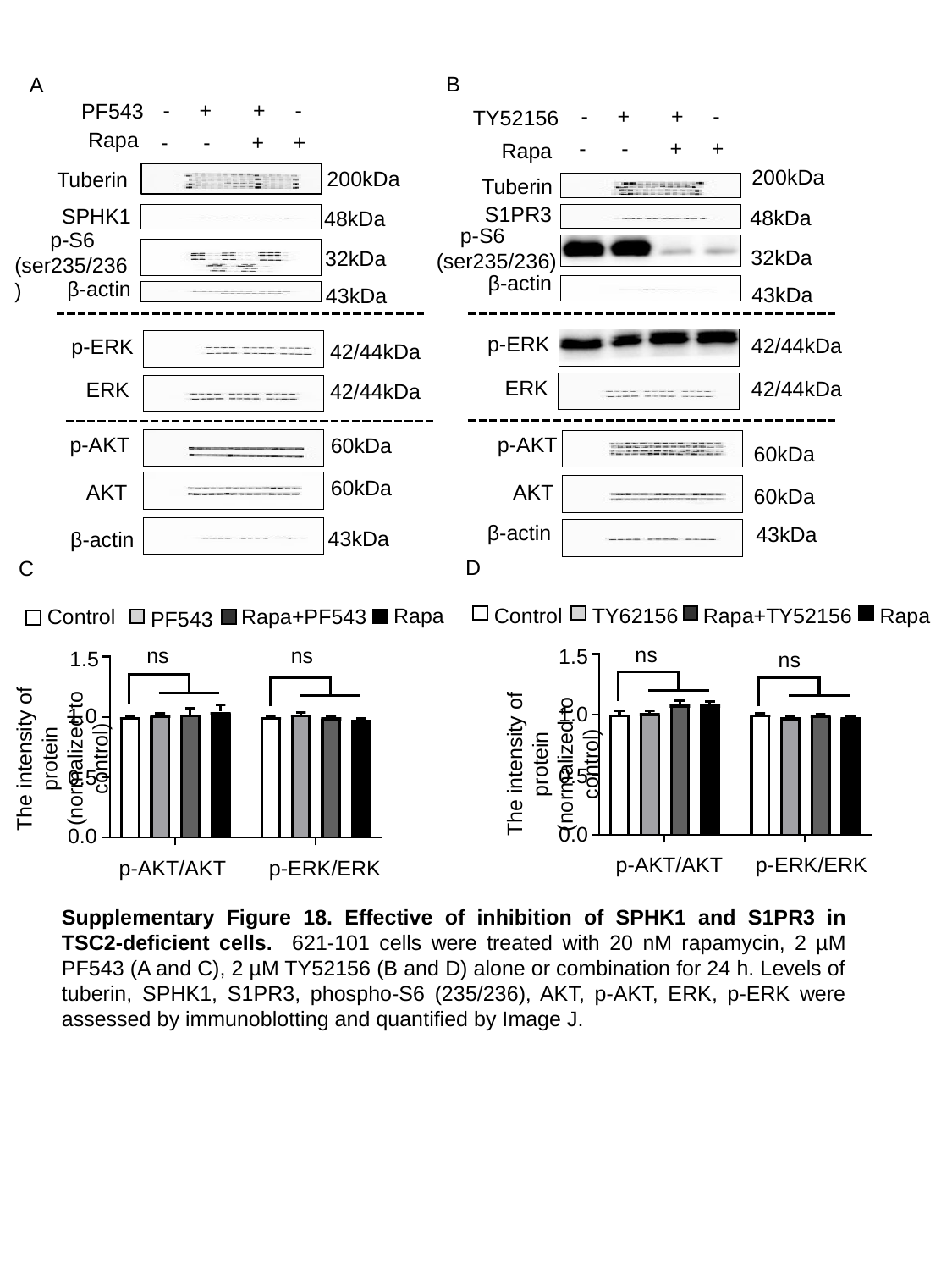

B
A
- + + -
PF543
Rapa
- - + +
Tuberin
SPHK1
48kDa
 p-S6
(ser235/236)
32kDa
β-actin
43kDa
200kDa
- + + -
TY52156
- - + +
Rapa
200kDa
Tuberin
S1PR3
48kDa
 p-S6
(ser235/236)
32kDa
β-actin
43kDa
p-ERK
42/44kDa
ERK
42/44kDa
p-AKT
60kDa
AKT
60kDa
β-actin
43kDa
p-ERK
42/44kDa
ERK
42/44kDa
p-AKT
60kDa
60kDa
AKT
43kDa
β-actin
D
C
Control
TY62156
Rapa+TY52156
Rapa
ns
1.5
ns
The intensity of protein
(normalized to control)
1.0
0.5
0.0
p-AKT/AKT
p-ERK/ERK
Rapa
Rapa+PF543
Control
PF543
The intensity of protein
(normalized to control)
p-AKT/AKT
p-ERK/ERK
ns
ns
1.5
1.0
0.5
0.0
Supplementary Figure 18. Effective of inhibition of SPHK1 and S1PR3 in TSC2-deficient cells. 621-101 cells were treated with 20 nM rapamycin, 2 µM PF543 (A and C), 2 µM TY52156 (B and D) alone or combination for 24 h. Levels of tuberin, SPHK1, S1PR3, phospho-S6 (235/236), AKT, p-AKT, ERK, p-ERK were assessed by immunoblotting and quantified by Image J.

## Slide 10
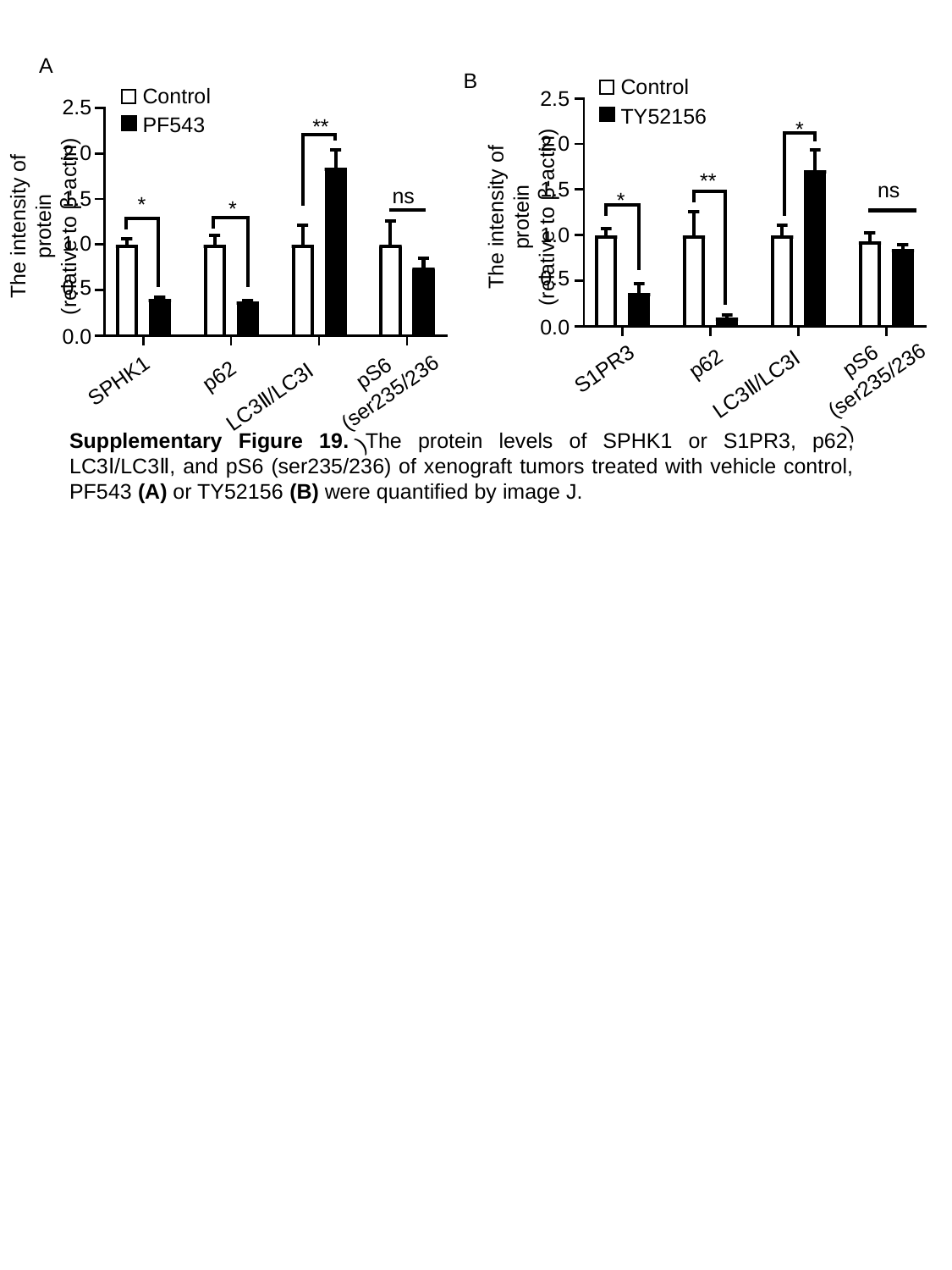

A
B
Control
2.5
TY52156
The intensity of protein
(relative to ꞵ-actin)
 *
2.0
 **
1.5
 ns
 *
1.0
0.5
0.0
 pS6
(ser235/236)
p62
S1PR3
LC3Ⅱ/LC3Ⅰ
Control
2.5
PF543
 **
The intensity of protein
(relative to ꞵ-actin)
2.0
 ns
1.5
 *
 *
1.0
0.5
0.0
 pS6
(ser235/236)
p62
SPHK1
LC3Ⅱ/LC3Ⅰ
Supplementary Figure 19. The protein levels of SPHK1 or S1PR3, p62, LC3Ⅰ/LC3Ⅱ, and pS6 (ser235/236) of xenograft tumors treated with vehicle control, PF543 (A) or TY52156 (B) were quantified by image J.
