# Supplementary material for: Targeting SPHK1/S1PR3-regulated S-1-P metabolic disorder triggers autophagic cell death in pulmonary lymphangiomyomatosis (LAM)
Source: Cell Death Dis. 2022 Dec 21;13(12):1065. doi: 10.1038/s41419-022-05511-3 (PMC9772321; doi:10.1038/s41419-022-05511-3)
Supplement: Supplementary file 2 — Supplementary Information [file 41419_2022_5511_MOESM2_ESM.docx]

**Supplementary Information**

**ELISA assay**

621-101 and 621-103 cells, 621-103-siRNA-*vector* and 621-103-siRNA-*TSC2* cells or HEK293T-siRNA-*vector and* HEK293T-siRNA-*TSC2* cells were seeded in 6-well plate and collected after 48 hours. The level of S1P was measured using human S1P ELISA kit (Blue Gene, E01S0323) and the results were calculated according to the protocol.

**Expression array analysis**

Public available expression array data was re-analysis using online GEO2R tool (GEO number #GSE16944, Lee et al, 2009, PMID: 19395678). Originally, gene expression data were obtained in triplicate on three groups of cells: TSC2-deficient (TSC2-) cells treated with 10 nM rapamycin (group A), vehicle-treated TSC2- cells (group B), and TSC2-addback (TSC2+) cells (group C). The gene expression levels on each array were normalized to a median expression level of 1.0 using the CodeLink software. To focus on genes expressed well above background, genes were excluded from consideration if no sample had an expression level of at least 2. Coefficients of variation, intergroup P values (Student's t test), and gene ratios of two-group comparisons were calculated using Microsoft Excel, and then imported to Microsoft Access for further analysis. To identify differentially expressed genes, a within-group coefficient of variation of less than 0.2 was required to exclude highly variable genes obtained, and then a P value of less than 0.05 for the Student's t test for the average expression between any pair of groups.

To identify genes related loss of TSC2 but independent of mTORC1 activation, following criteria were selected: (1) expression ratio of group A: group C greater than 2, with a P value less than 0.05; (2) expression ratio of group B: group C greater than 2 with a P value less than 0.05; and (3) group A and group B expression not significantly different (P > 0.05). And a subset of these genes was also identified whose expression levels were increased more than fivefold in the TSC2-deficient cells with or without rapamycin treatment (groups A and B) in comparison to TSC2+ cells (group C).

The two-sided Student's t test was used to compare expression between pairs of groups. The simple Bonferroni correction was used to correct for multiple comparisons in gene expression data for the sphingosine metabolic enzymes.

The gene expression levels in all three groups were compared in a pair-wise fashion. The TSC2-deficient cells with or without rapamycin treatment (groups A and B) were compared with each other to identify rapamycin-dependent genes, and both groups A and B were compared with the TSC2+ cells (group C) to identify TSC2-related genes.

According to above expression array analysis method and criteria, we performed sphingosine metabolic enzymes profiling using expression array of #Gse16944.

**The list of human si*TSC2* sequences**

Line 1: Sequence (5’to 3’): CUUGUCUGGACAUGAUGGCUCGAUA

Line 2: Sequence (5’to 3’): UAUCGAGCCAUCAUGUCCAGACAGG

Line 3: Sequence (5’to 3’): GCUGGUGAACUUGGUCAAAUUCAAU

**The list of human si*S1PR3* sequences**

#1: Sequence (5’to 3’): CCAAGAAGUACAUUGCCUUTT

AAGGCAAUGUACUUCUUGGTT

#2: Sequence (5’to 3’): GCAUCGCUUACAAGGUCAATT

UUGACCUUGUAAGCGAUGCTT

#3: Sequence (5’to 3’): CCGUGCUCUUCUUGGUCAUTT

AUGACCAAGAAGAGCACGGTT

**The list of human shSPHK1 sequences:**

#1: GCAGCTTCCTTGAACCATTAT

#2: CCTGACCAACTGCACGCTATT

**The list of qPCR primers and their sequences**

Human TSC2

F: GCACCTCTACAGGAACTTTGCC, R: GCACCTGATGAACCACATGGCT

Human ASAH1

F: ATTGGCCCCAGCCTACTTTAT, R: CCCTGCTTAGCATCGAGTTCAT.

Human SPHK1

F: GCTGGCAGCTTCCTTGAACCAT, R: GTGTGCAGAGACAGCAGGTTCA

Human S1PR1

F: TCTGCGGGAAGGGAGTATGT, R: CGATGGCGAGGAGACTGAA

Human S1PR2

F: CACCTGGCGGTACAAAGAAT, R: GTCAAGTGGCAGCTGATGAA

Human S1PR3

F: TCTCAGCCTTCATCCATTAACTCTAC, R: AGGGAGCCTTATGTCATACCACAA

Human S1PR4

F: GAGTCATACCCACAGTTGC, R: CAGTGTGATGTTCAGCAGG

Human S1PR5

F: CCCTGTGACTACCAAAGTTCA, R: TACTCCTGCCAGCCCTTA

Human SGPL

F: GAACACTGCCATGCTCGTCTGT, R: GATGAGGAAGCCTCCCAGACAA

Human ATG5

F: AGAAGCTGTTTCGTCCTGTGG, R: AGGTGTTTCCAACATTGGCTC

Human β-actin

F: CACCATTGGCAATGAGCGGTTC, R: AGGTCTTTGCGGATGTCCACGT

Rat TSC2

F: AGCCTGCCTCTGTTCATTATC, R: GGCCTAGATGGGTACCTAGAA

Rat ASAH1

F: GCCCAATGGGTAGGGTTTAT, R: TGTCCTGTATGCTGGCTGAT

Rat SPHK1

F: TTAAACTGATGCTCACCGAACGG, R: CACGAGGTGGTGAATGGGCT

Rat α-tubulin

F: GACCTGGAACCCACAGTTATT, R: ATCTTCCTTGCCTGTGATGAG

Mouse TSC2

F: TGCCGCAGCATCAGTGTATC, R: TGCCAGGAGGAACTCTCCC

Mouse ASAH1

F: CGTGGACAGAAGATTGCAGAA, R: TGGTGCCTTTTGAGCCAATAAT

Mouse SPHK1

F: GGTGAATGGGCTAATGGAACG, R: CTGCTCGTACCCAGCATAGTG

Mouse GAPDH

F: CATCACTGCCACCCAGAAGACTG, R: ATGCCAGTGAGCTTCCCGTTCAG
