# Supplementary material for: Targeting SPHK1/S1PR3-regulated S-1-P metabolic disorder triggers autophagic cell death in pulmonary lymphangiomyomatosis (LAM)
Source: Cell Death Dis. 2022 Dec 21;13(12):1065. doi: 10.1038/s41419-022-05511-3 (PMC9772321; doi:10.1038/s41419-022-05511-3)
Supplement: Supplementary file 3 — Supplemental material (WB) [file 41419_2022_5511_MOESM3_ESM.pptx]

## Slide 1
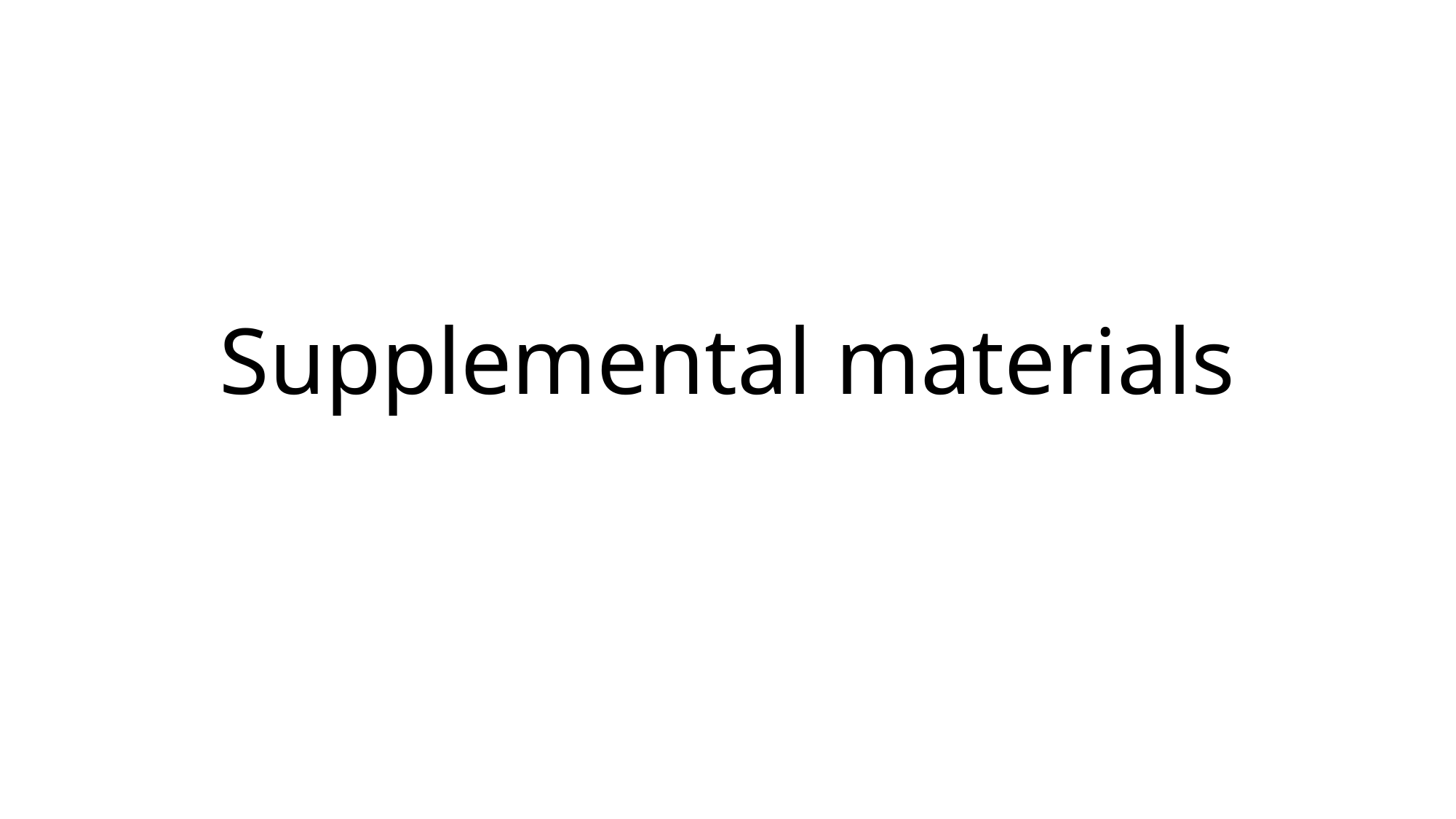

# Supplemental materials

## Slide 2
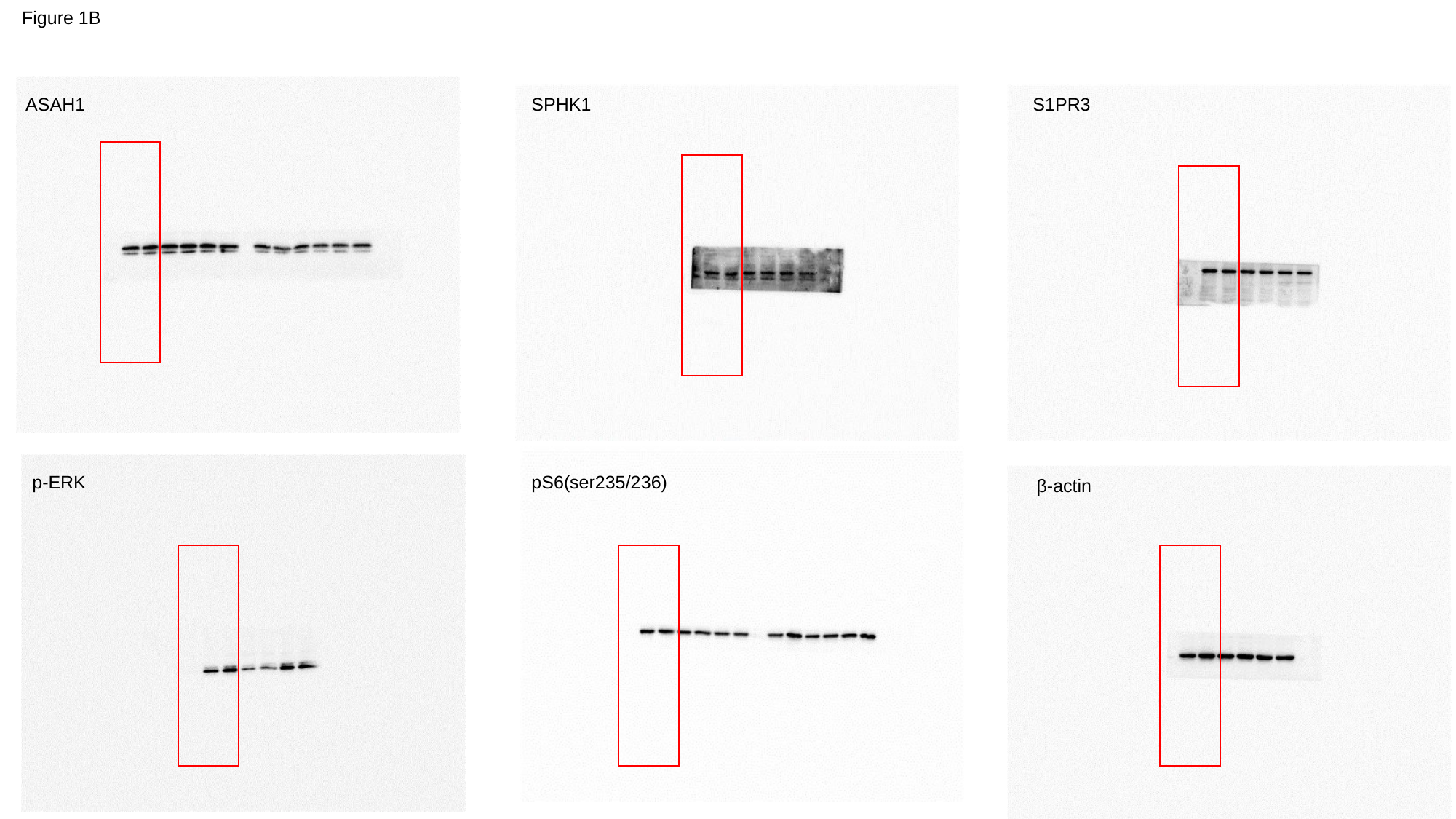

Figure 1B
ASAH1
SPHK1
S1PR3
p-ERK
pS6(ser235/236)
β-actin

## Slide 3
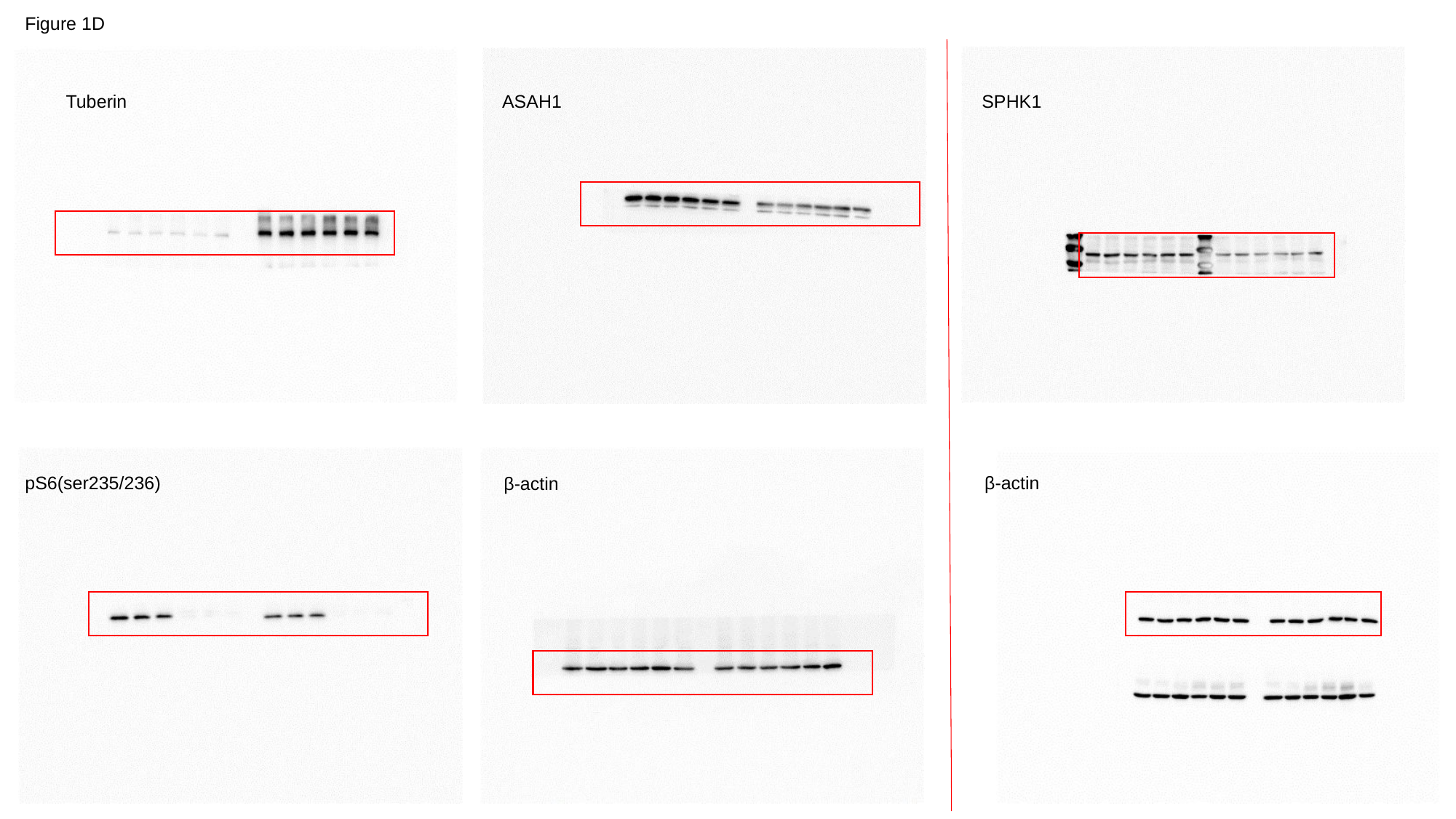

Figure 1D
Tuberin
ASAH1
SPHK1
pS6(ser235/236)
β-actin
β-actin

## Slide 4
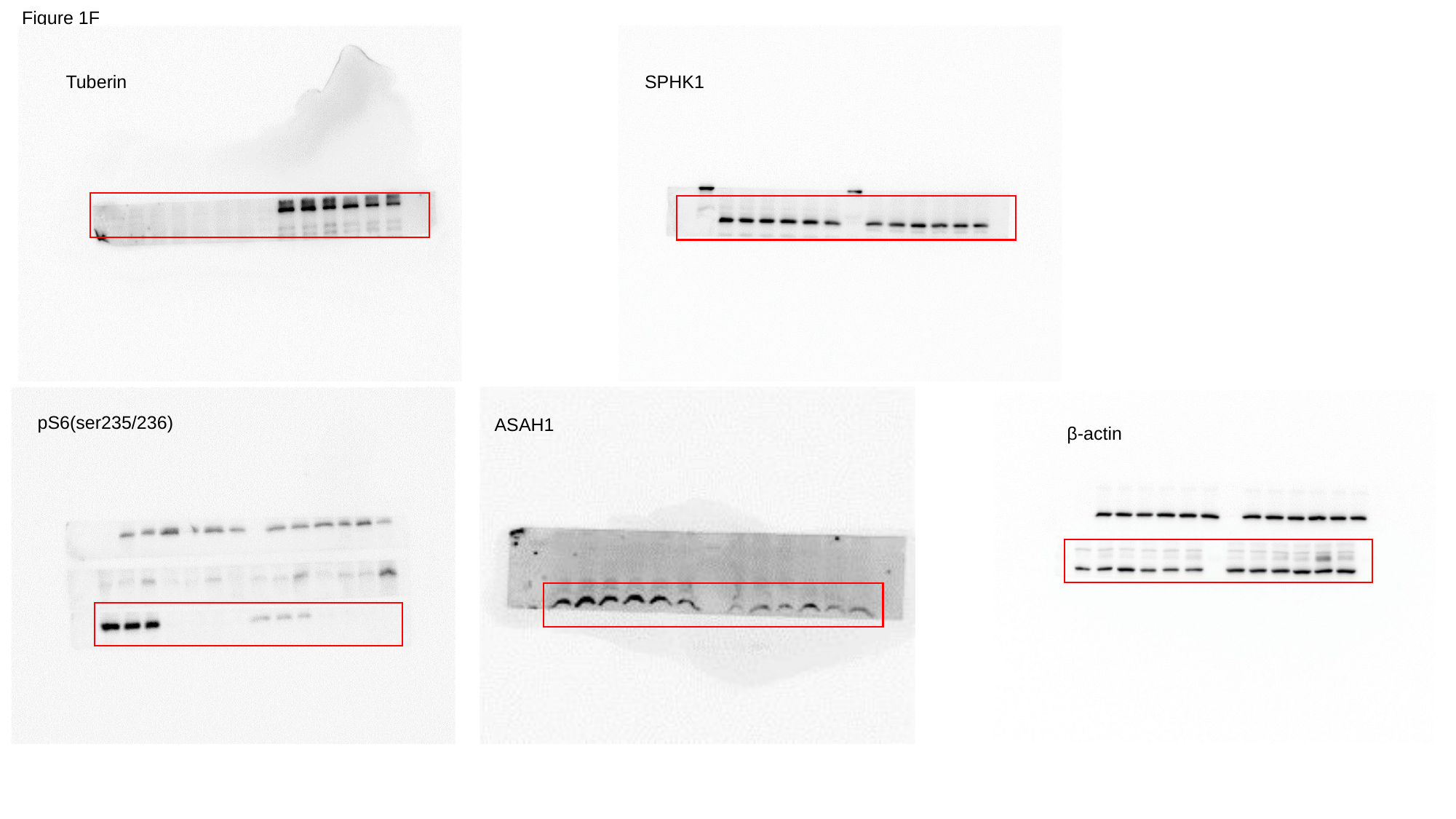

Figure 1F
Tuberin
SPHK1
pS6(ser235/236)
ASAH1
β-actin

## Slide 5
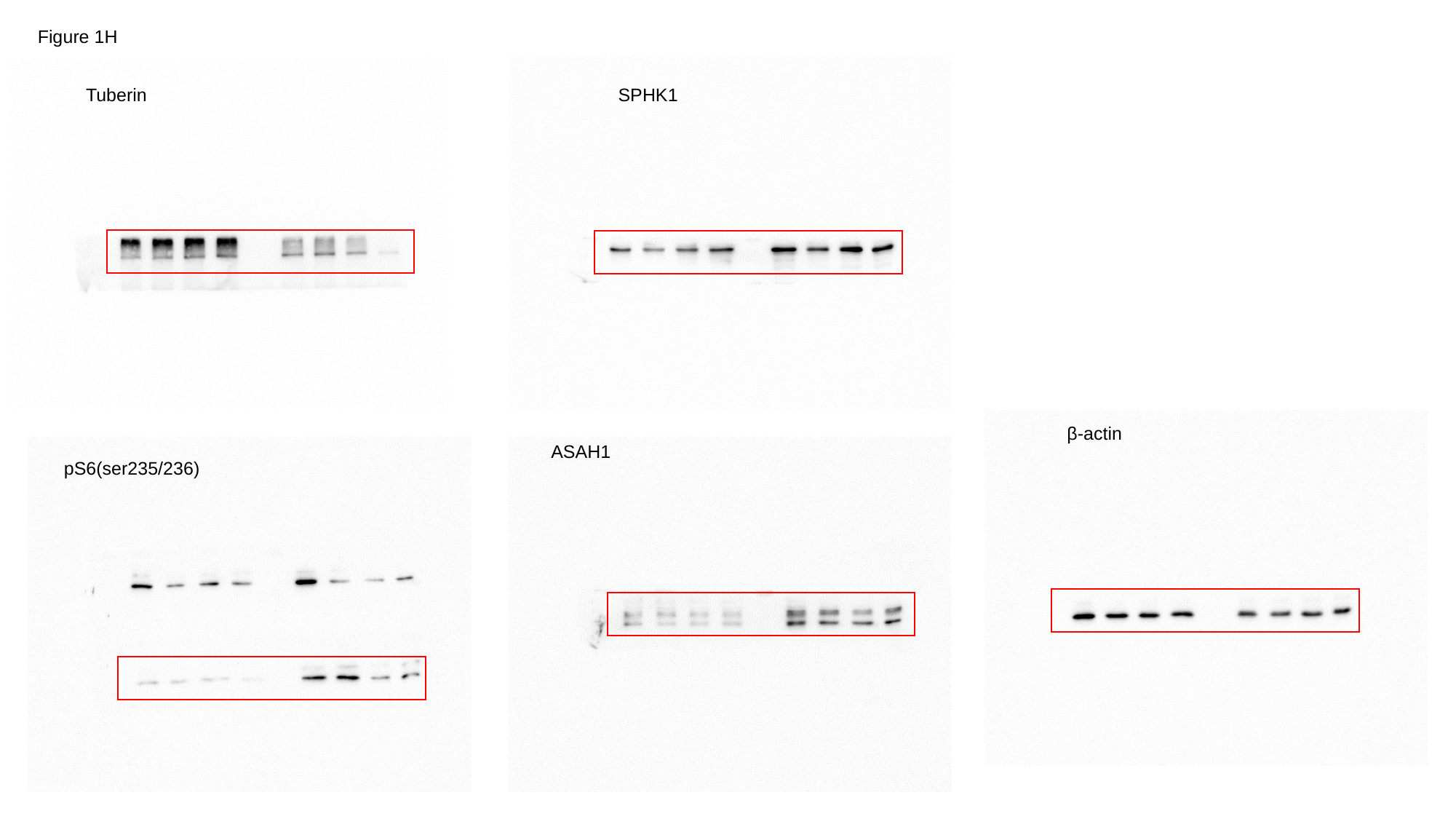

Figure 1H
Tuberin
SPHK1
β-actin
ASAH1
pS6(ser235/236)

## Slide 6
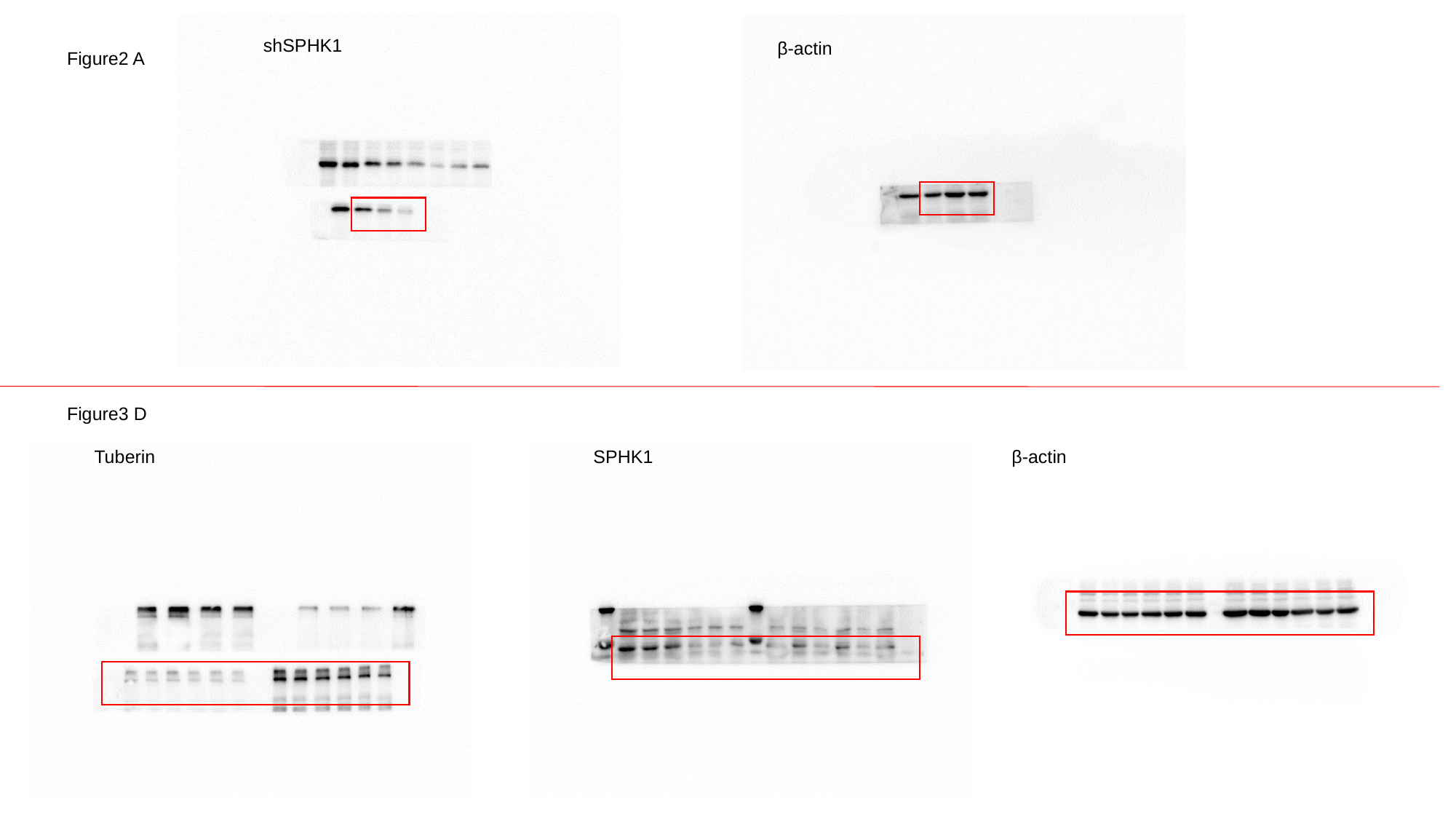

shSPHK1
β-actin
Figure2 A
Figure3 D
Tuberin
SPHK1
β-actin

## Slide 7
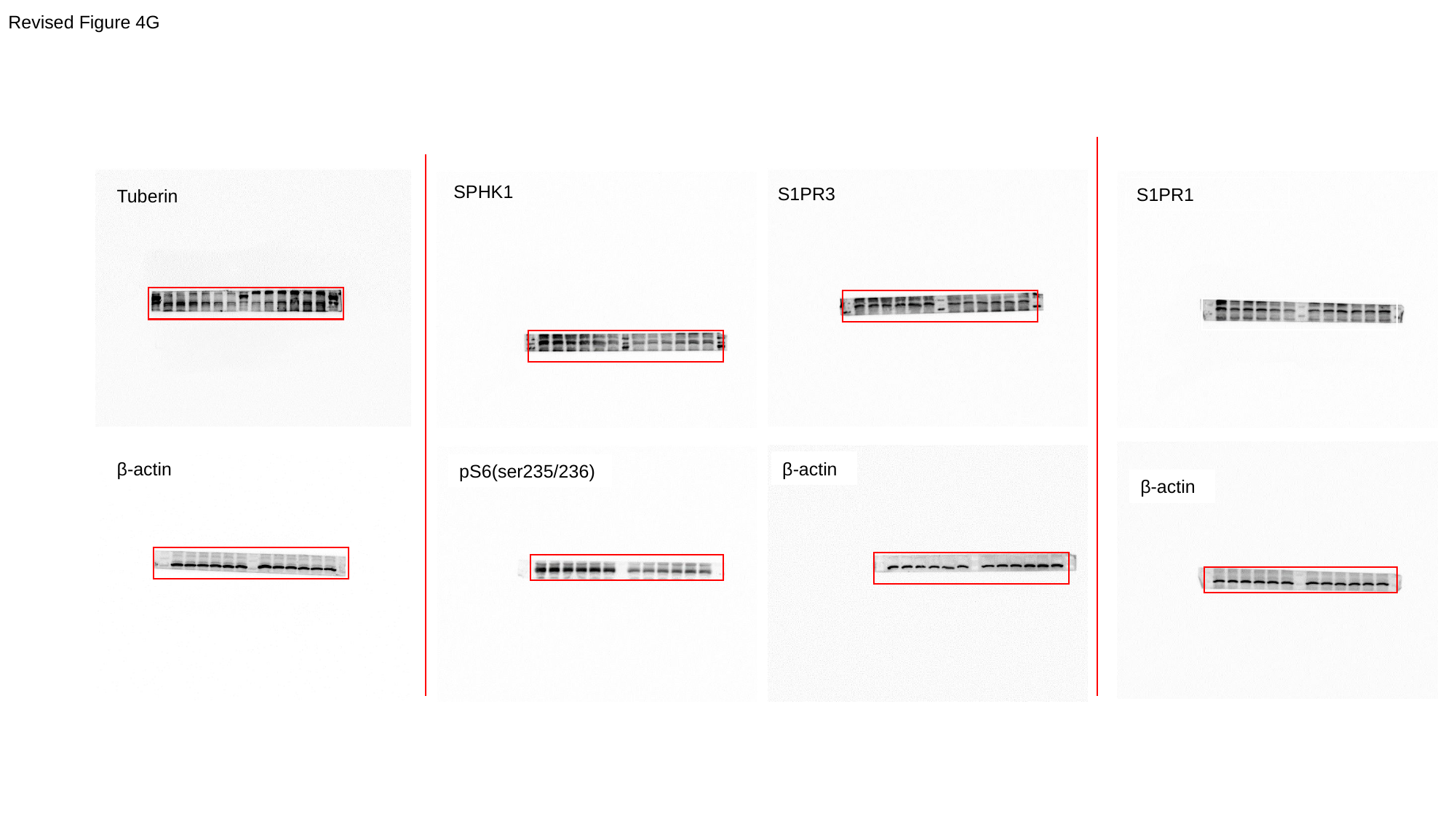

Revised Figure 4G
Tuberin
S1PR3
SPHK1
S1PR1
β-actin
β-actin
pS6(ser235/236)
β-actin

## Slide 8
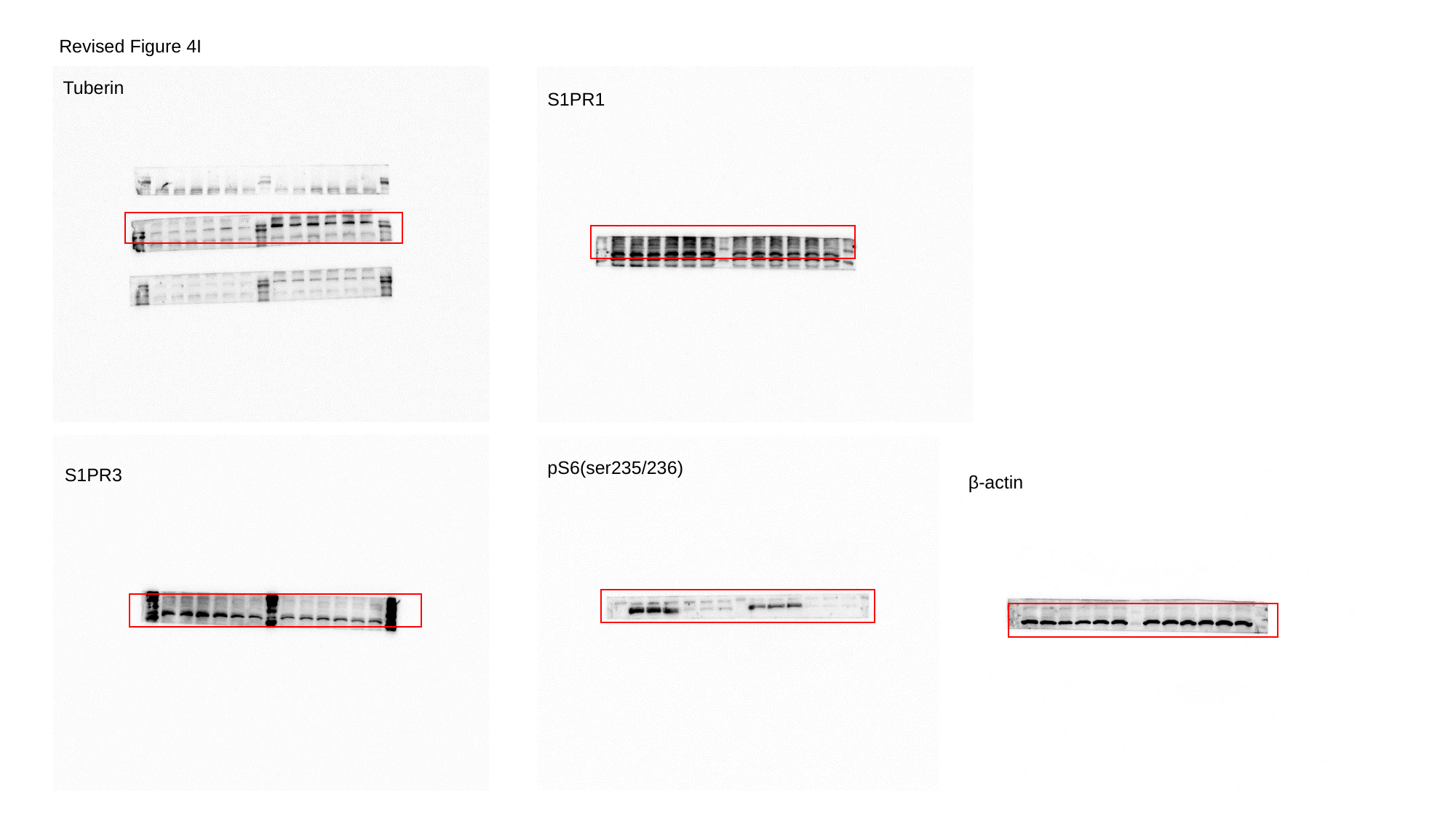

Revised Figure 4I
Tuberin
S1PR1
S1PR3
pS6(ser235/236)
β-actin

## Slide 9
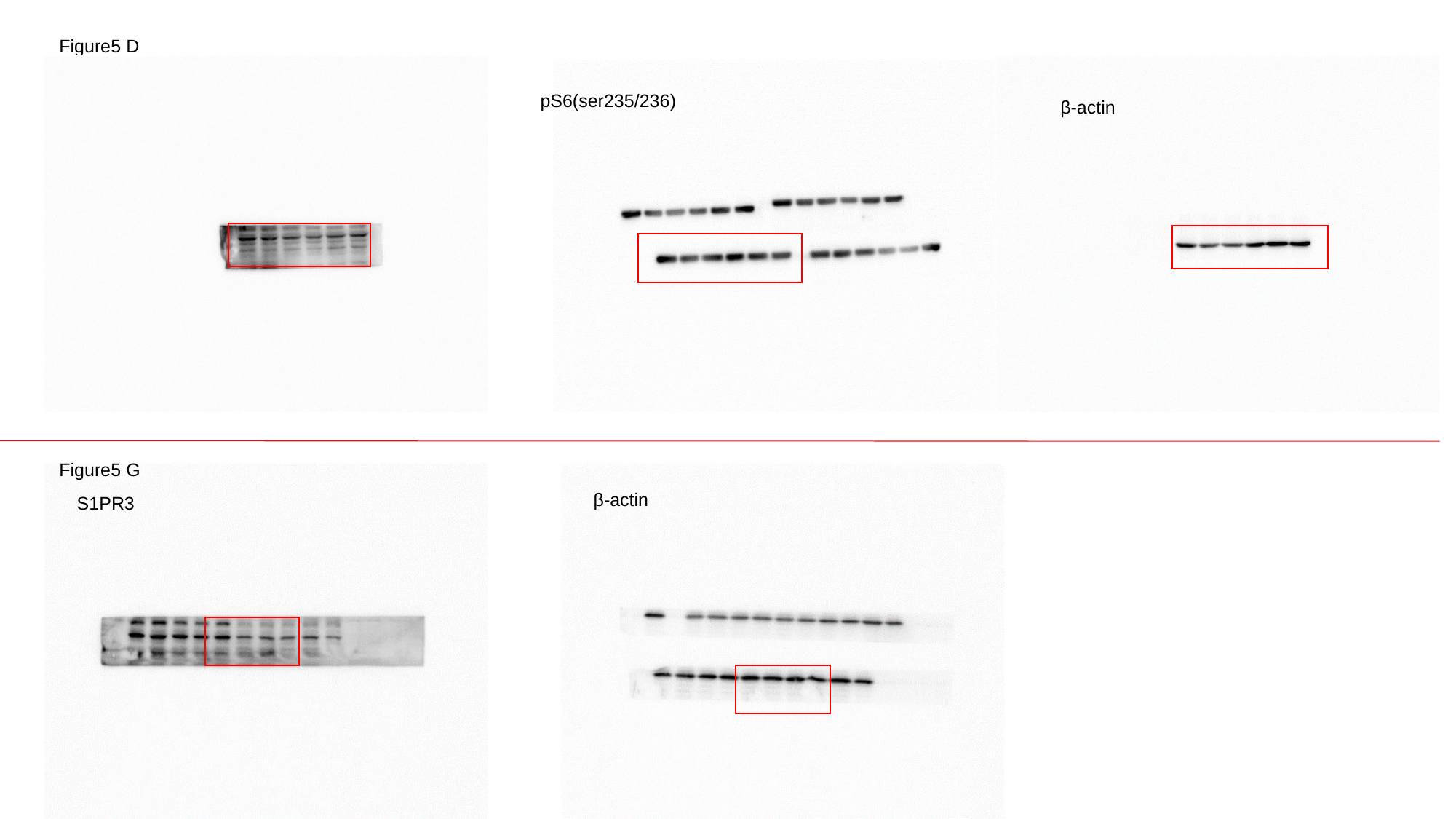

Figure5 D
pS6(ser235/236)
β-actin
S1PR3
Figure5 G
β-actin
S1PR3

## Slide 10
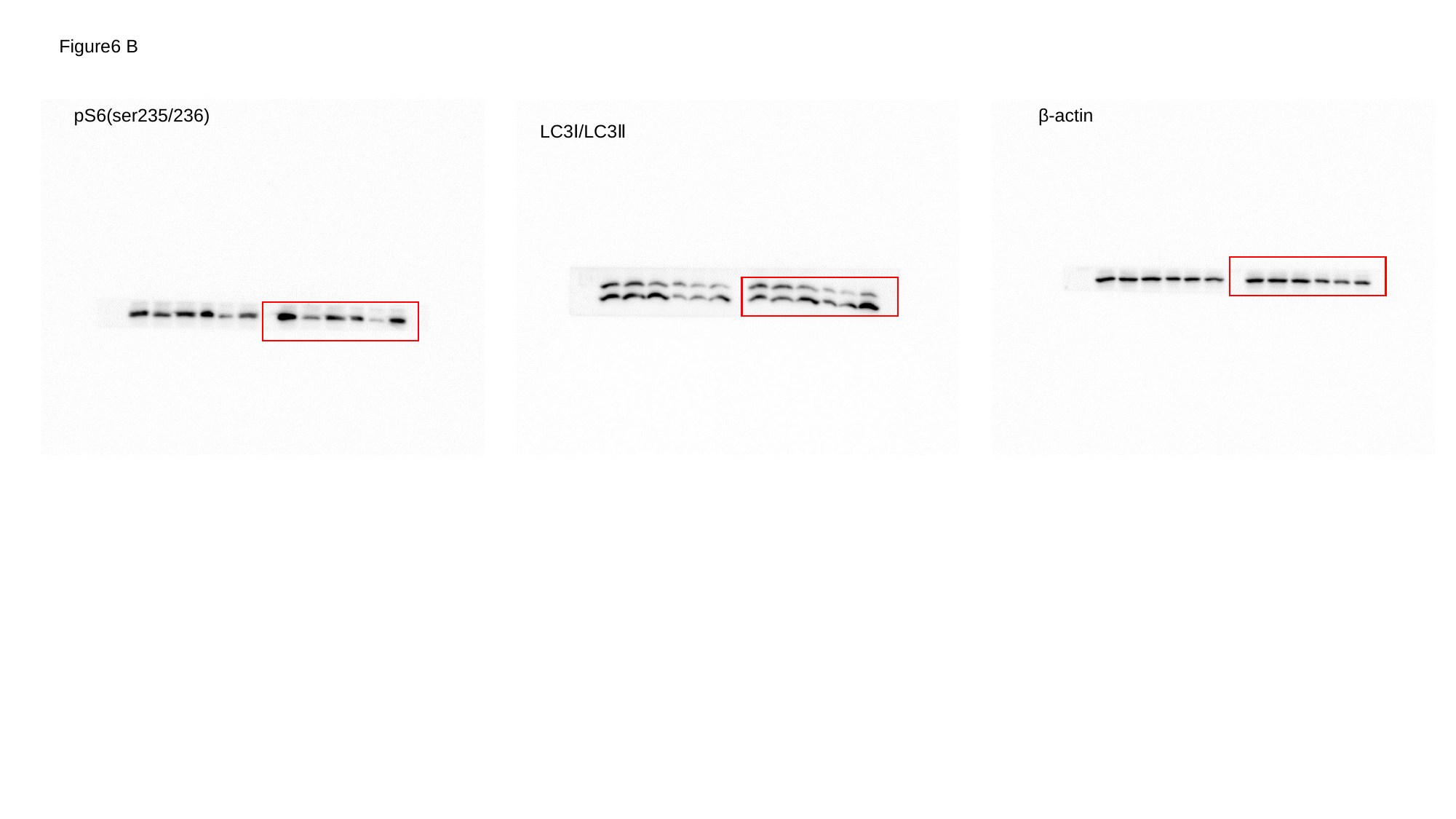

Figure6 B
pS6(ser235/236)
LC3Ⅰ/LC3Ⅱ
β-actin

## Slide 11
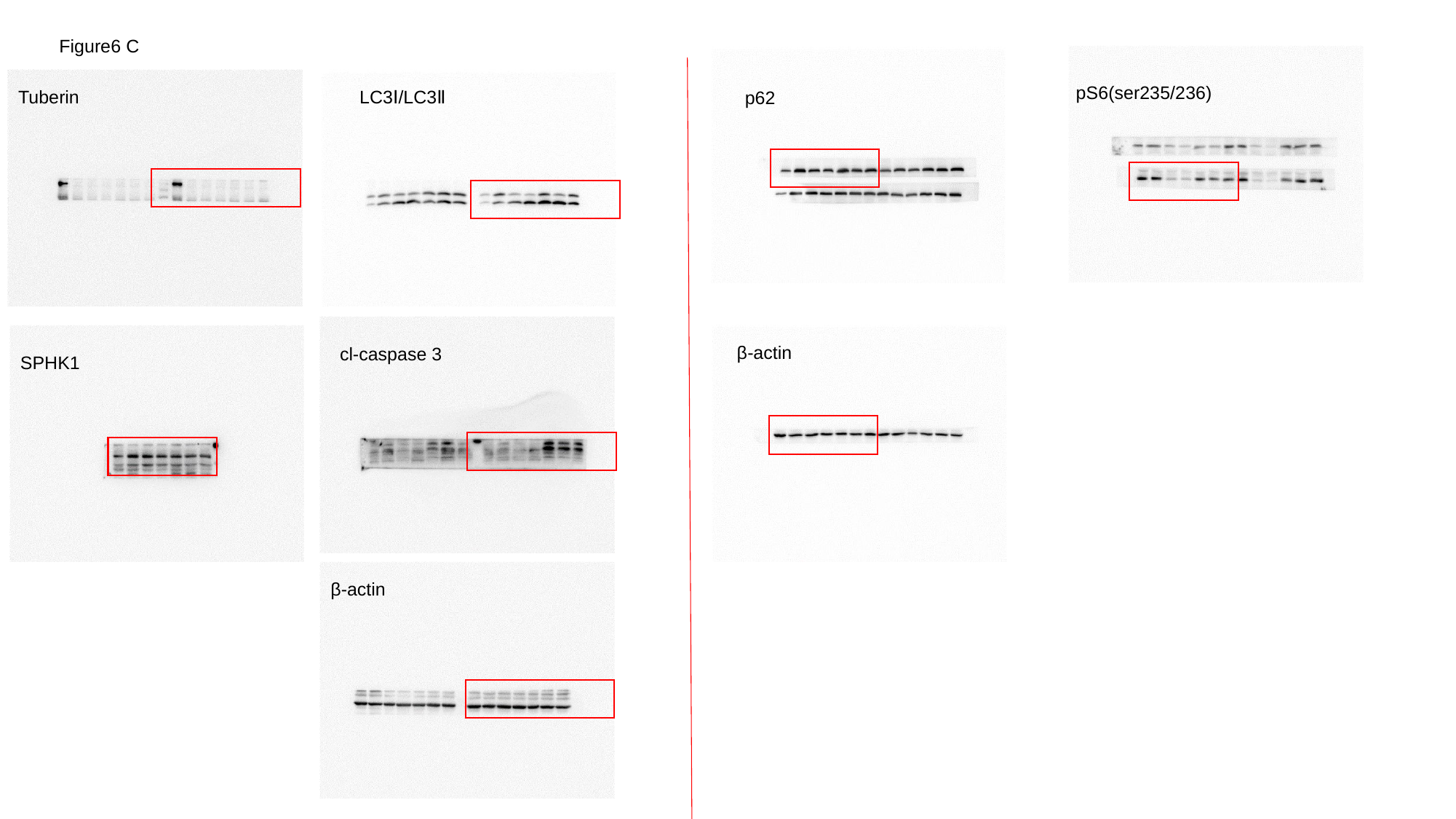

Figure6 C
pS6(ser235/236)
p62
LC3Ⅰ/LC3Ⅱ
Tuberin
cl-caspase 3
SPHK1
β-actin
β-actin

## Slide 12
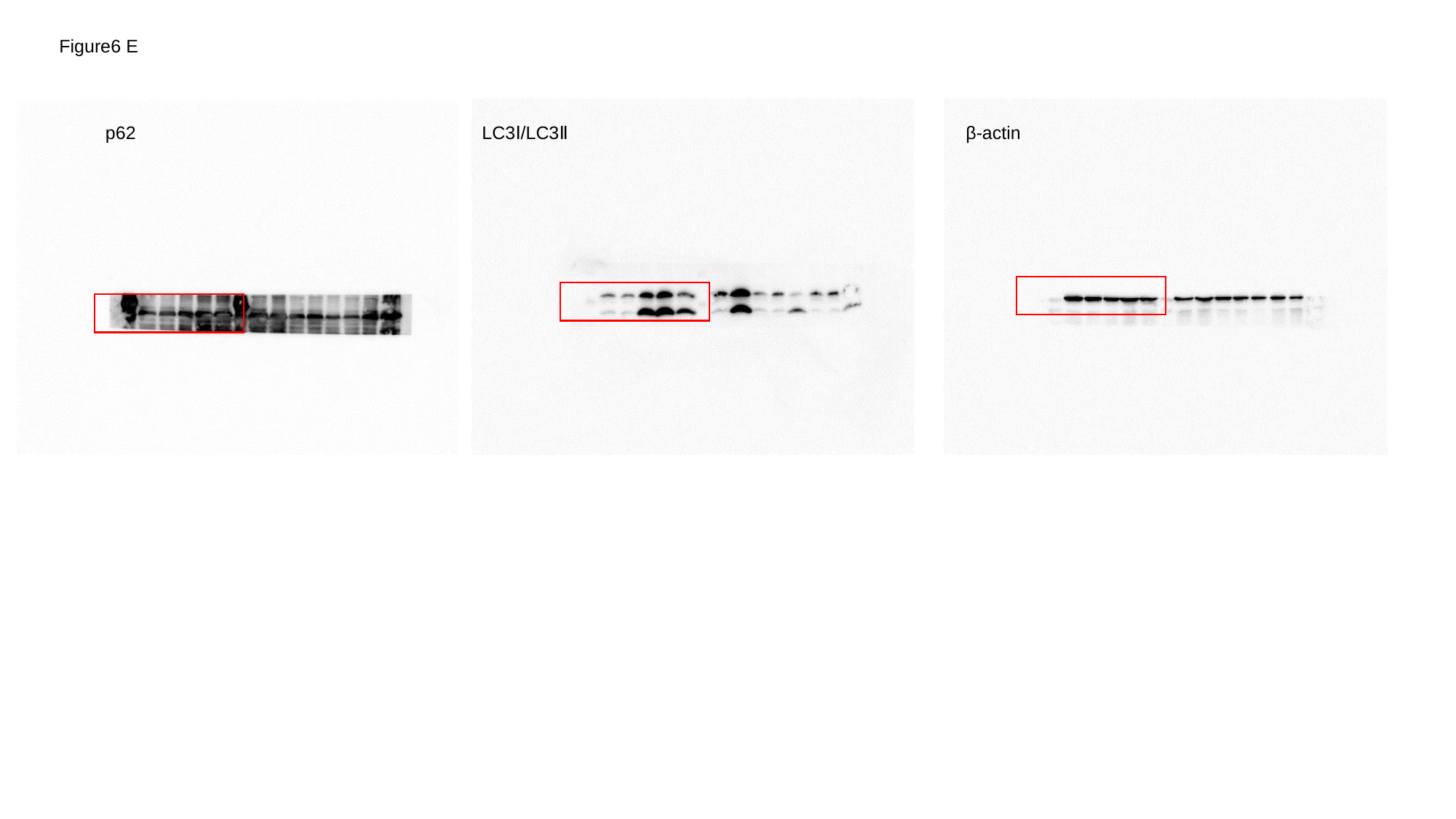

Figure6 E
p62
LC3Ⅰ/LC3Ⅱ
β-actin

## Slide 13
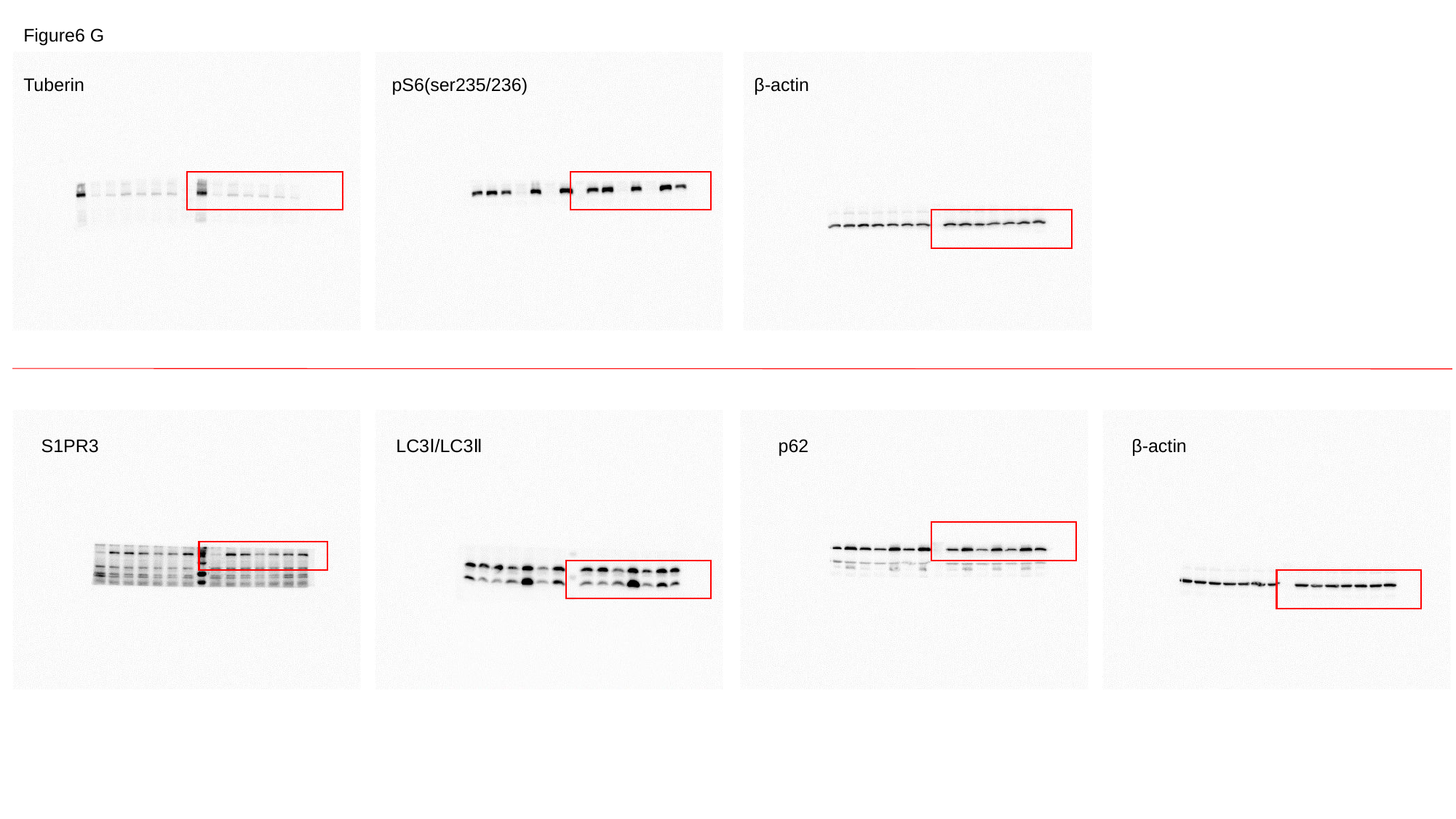

Figure6 G
Tuberin
pS6(ser235/236)
β-actin
S1PR3
LC3Ⅰ/LC3Ⅱ
p62
β-actin

## Slide 14
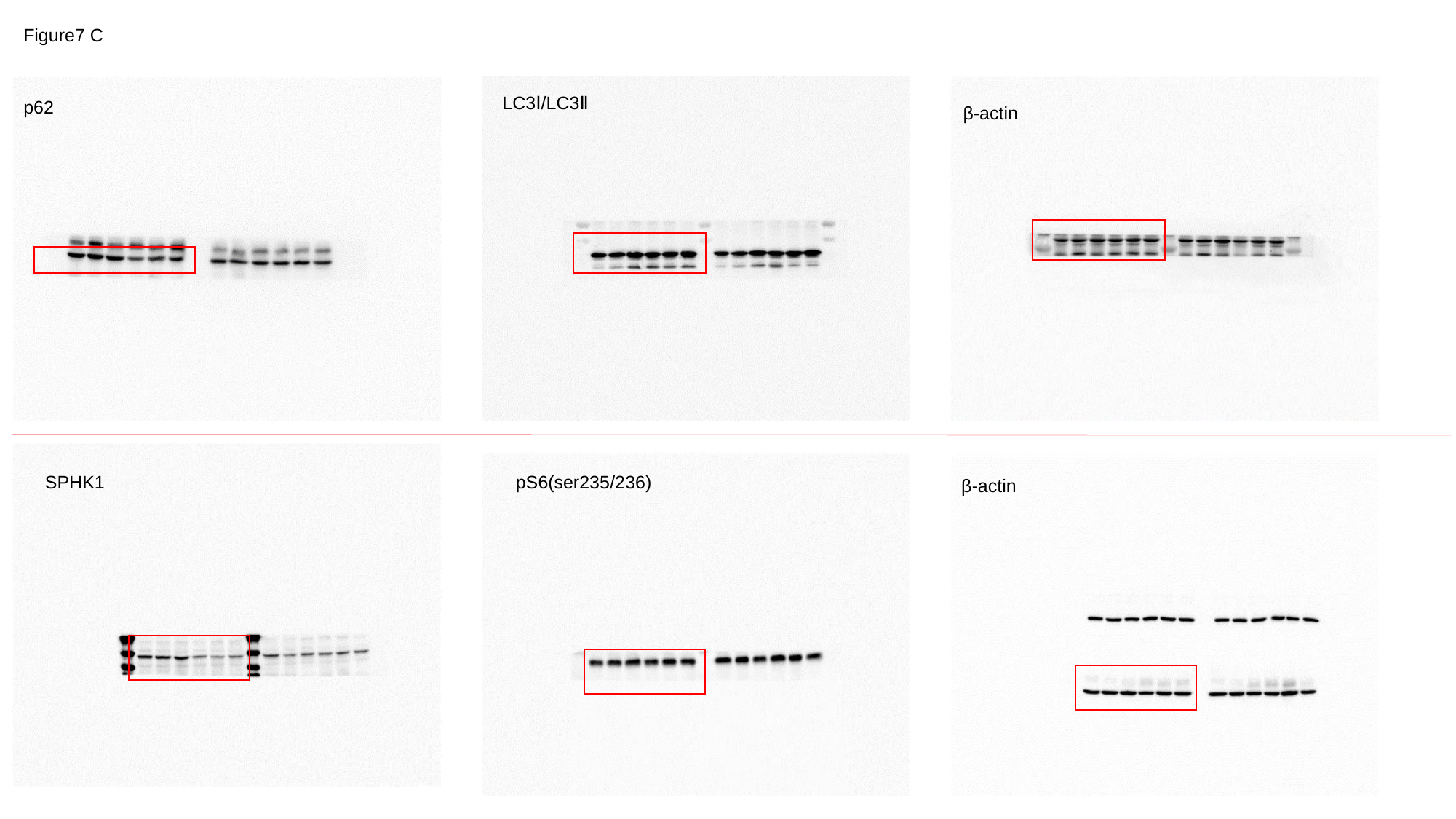

Figure7 C
p62
LC3Ⅰ/LC3Ⅱ
β-actin
SPHK1
pS6(ser235/236)
β-actin

## Slide 15
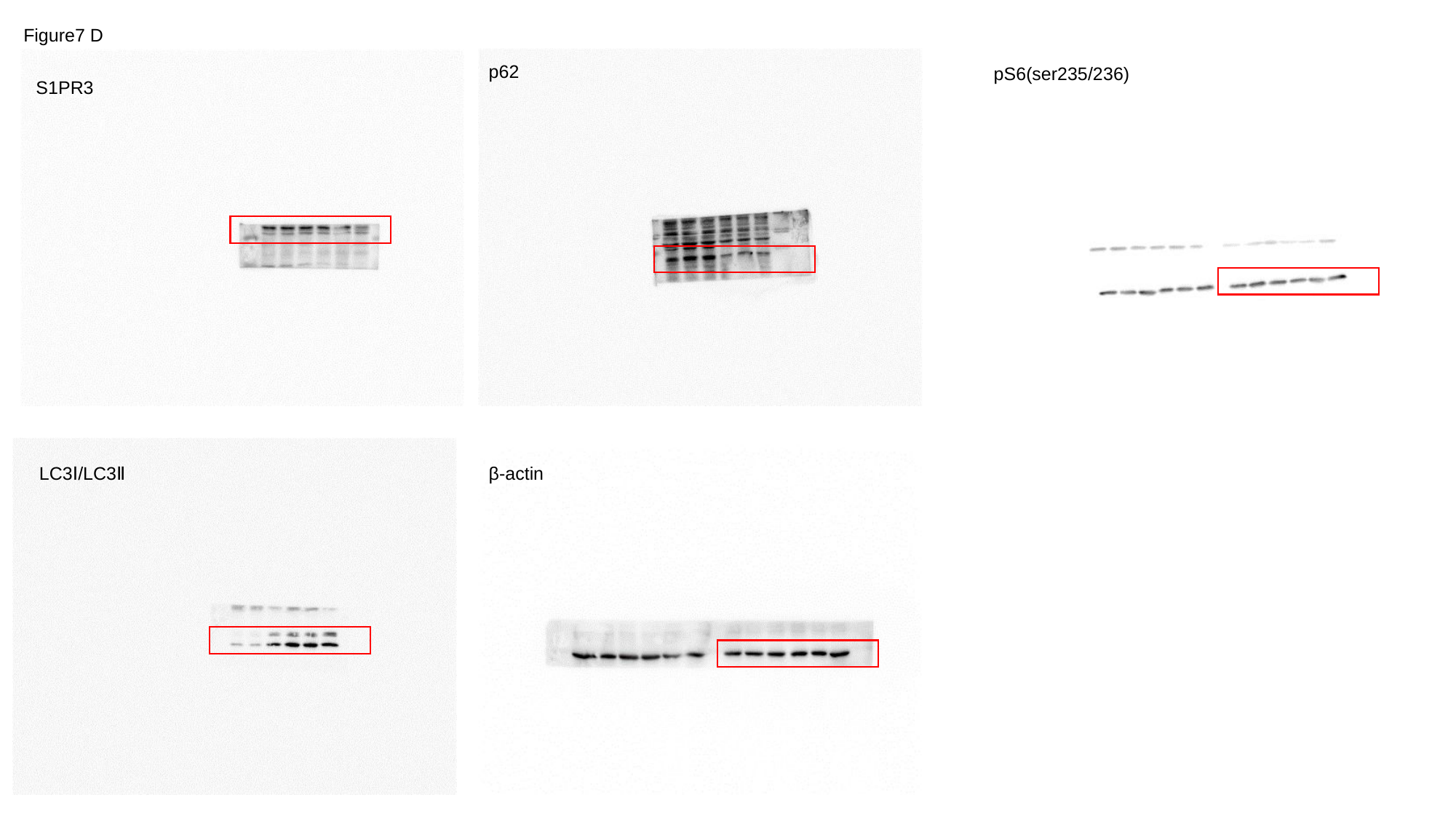

Figure7 D
p62
pS6(ser235/236)
S1PR3
β-actin
LC3Ⅰ/LC3Ⅱ

## Slide 16
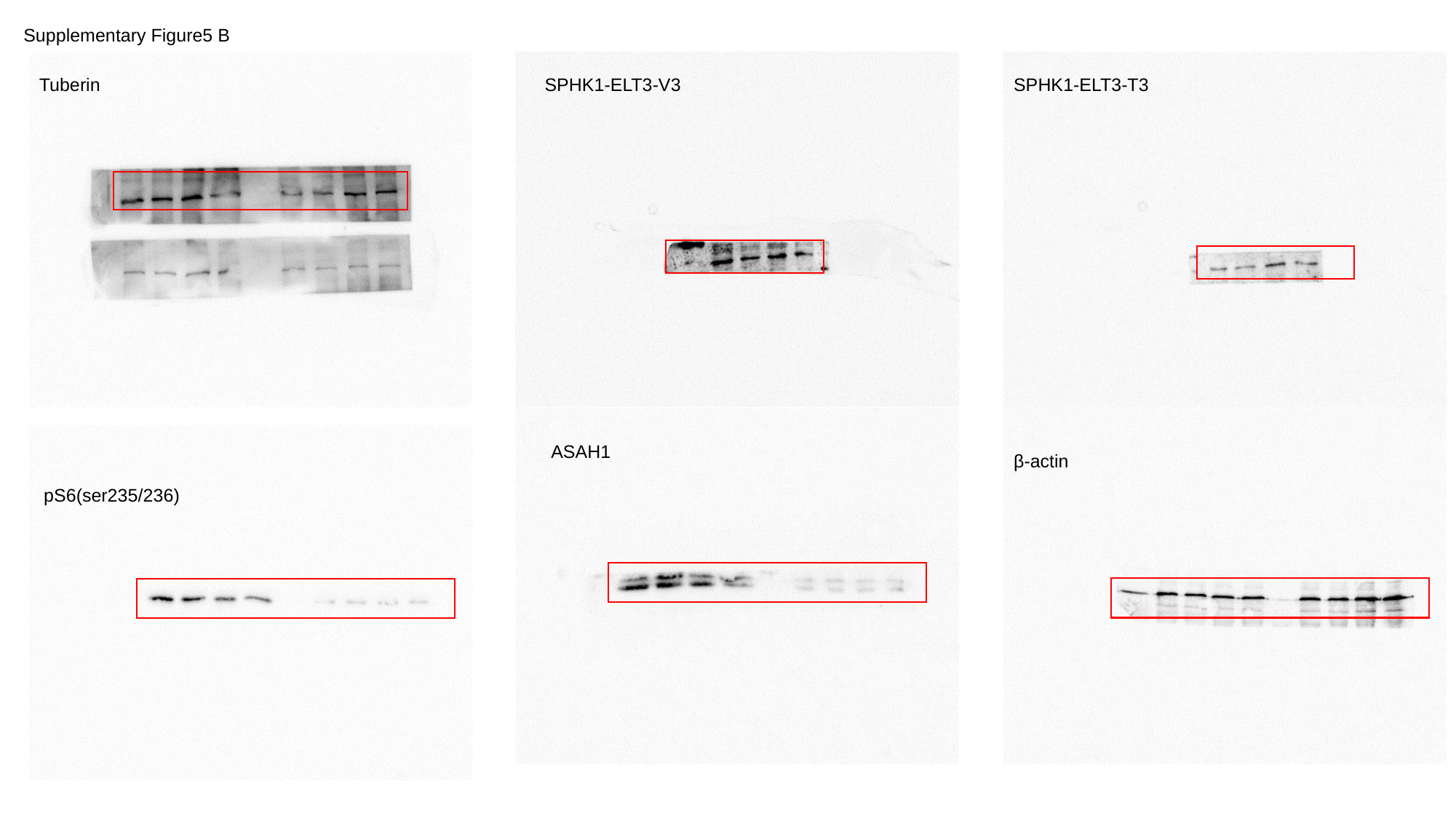

Supplementary Figure5 B
Tuberin
SPHK1-ELT3-V3
SPHK1-ELT3-T3
ASAH1
β-actin
pS6(ser235/236)

## Slide 17
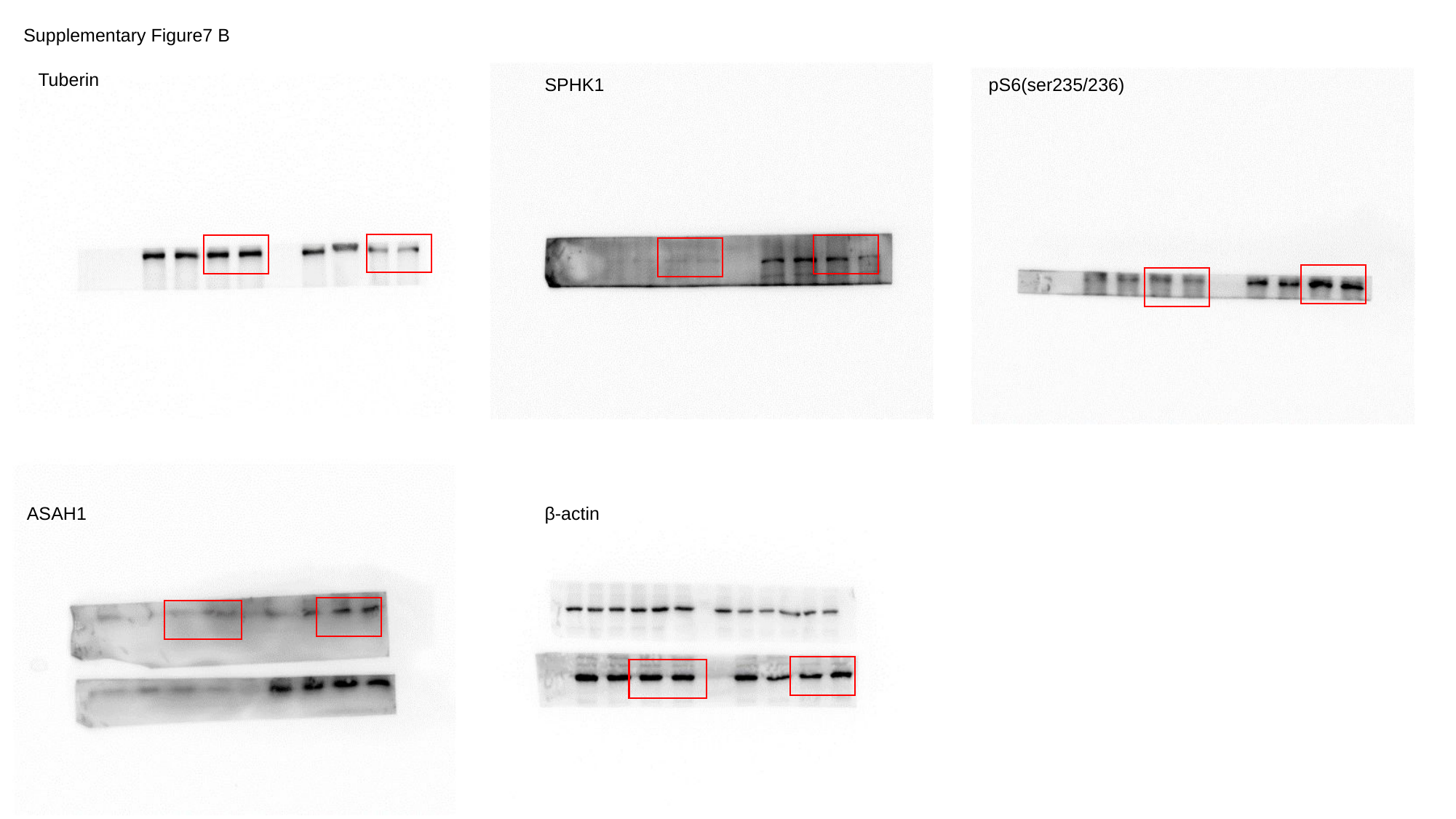

Supplementary Figure7 B
Tuberin
SPHK1
pS6(ser235/236)
β-actin
ASAH1

## Slide 18
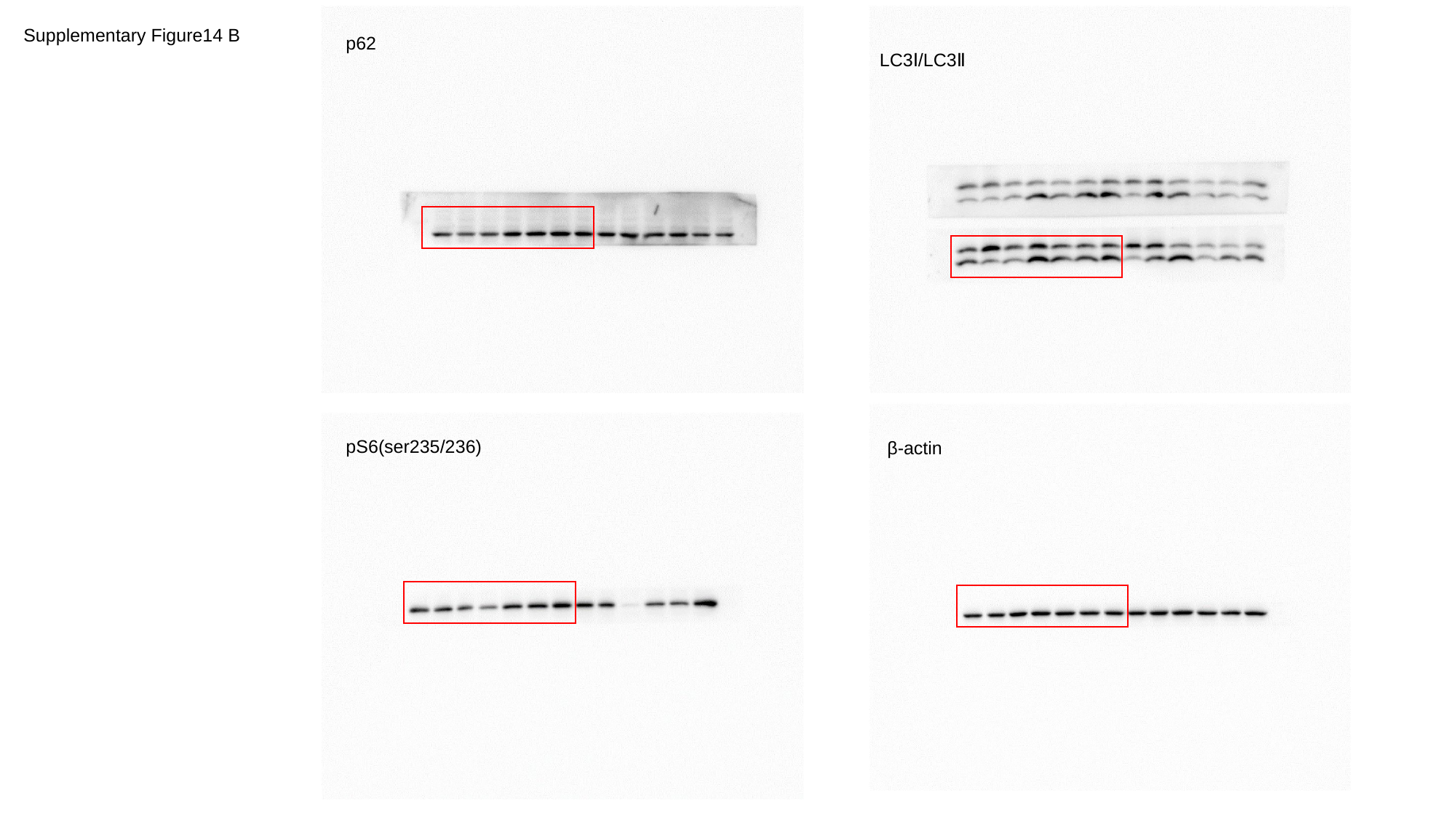

p62
LC3Ⅰ/LC3Ⅱ
Supplementary Figure14 B
β-actin
pS6(ser235/236)

## Slide 19
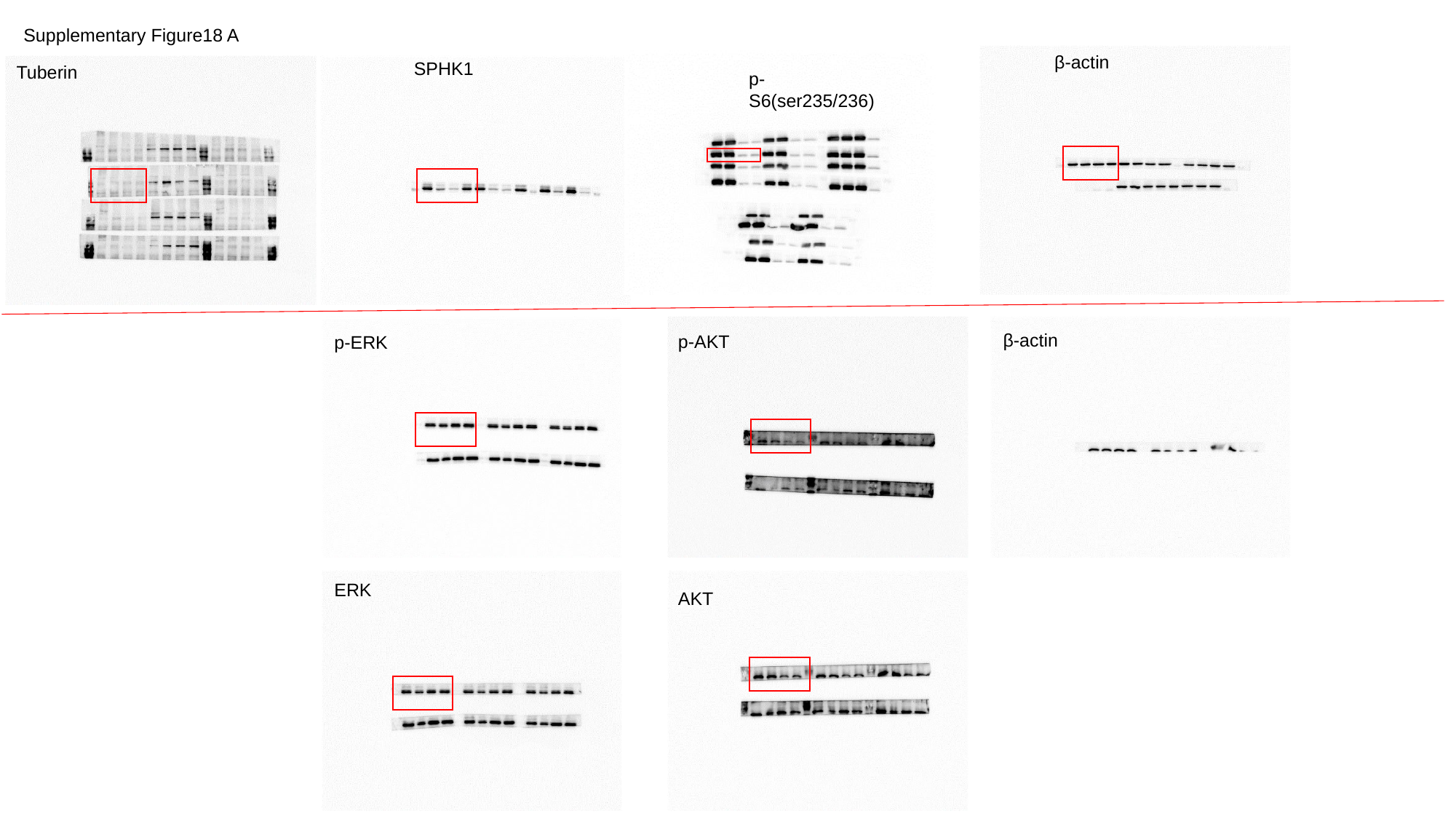

Supplementary Figure18 A
β-actin
p-S6(ser235/236)
SPHK1
Tuberin
p-ERK
p-AKT
β-actin
ERK
AKT

## Slide 20
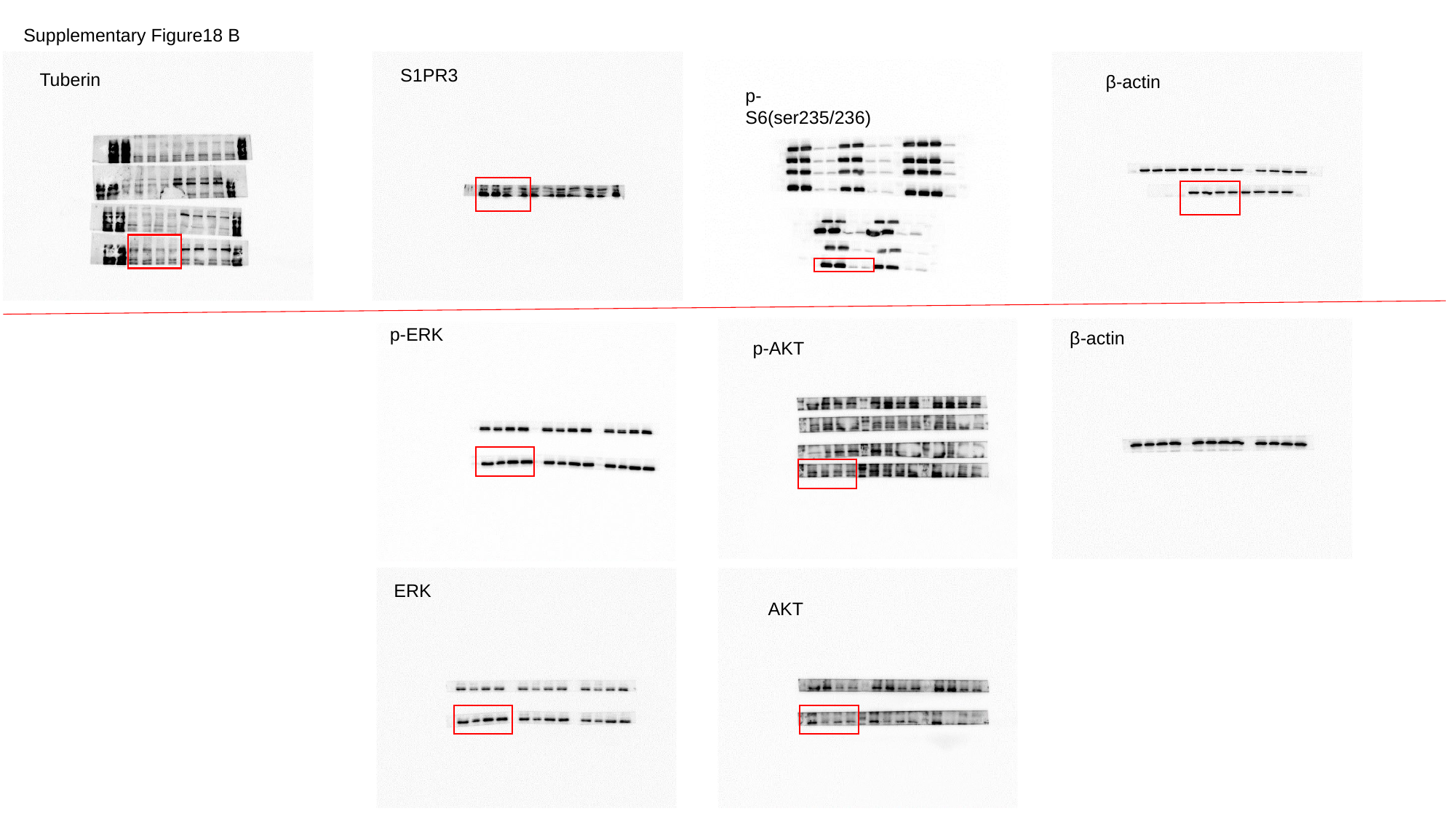

Supplementary Figure18 B
Tuberin
S1PR3
p-S6(ser235/236)
β-actin
p-ERK
p-AKT
β-actin
ERK
AKT
