# Supplementary material for: Targeting SPHK1/S1PR3-regulated S-1-P metabolic disorder triggers autophagic cell death in pulmonary lymphangiomyomatosis (LAM)
Source: Cell Death Dis. 2022 Dec 21;13(12):1065. doi: 10.1038/s41419-022-05511-3 (PMC9772321; doi:10.1038/s41419-022-05511-3)
Supplement: Supplementary file 4 — Author contributions [file 41419_2022_5511_MOESM4_ESM.docx]

**Author contributions**

F L, Y Z, Z L, Y Z, Q L, Y F, designed and performed in vitro studies, F L, Y Z, Y L, J Y and F Z designed and performed SPHK1 knockdown in vitro experiments. F L, L Y, Z L, Y Z, Y L, X H designed, performed and analyzed in vivo experiments. F L, Y Z, Q L, T L and Y Z performed and analyzed siRNA-S1PR3 experiments in vitro and in vivo. X P and X H provided guidance on data processing and writing. J J Yu and C L contributed to the study design, implementation and supervision of the study. Z F, D S, J J Y and C L contributed to the study design, implementation and supervision of the study, and review the manuscript. F L. and C L wrote the manuscript. All authors had full access to the data, and approved the final version of the manuscript.
